# The small molecule CBR-5884 inhibits the *Candida albicans* phosphatidylserine synthase

**DOI:** 10.1128/mbio.00633-24

**Published:** 2024-04-09

**Authors:** Yue Zhou, Gregory A. Phelps, Mikayla M. Mangrum, Jemma McLeish, Elise K. Phillips, Jinchao Lou, Christelle F. Ancajas, Jeffrey M. Rybak, Peter M. Oelkers, Richard E. Lee, Michael D. Best, Todd B. Reynolds

**Affiliations:** 1 Department of Microbiology, University of Tennessee, Knoxville, Tennessee, USA; 2 Department of Chemical Biology & Therapeutics, St. Jude Children’s Research Hospital, Memphis, Tennessee, USA; 3 Graduate School of Biomedical Sciences, St. Jude Children’s Research Hospital, Memphis, Tennessee, USA; 4 Department of Chemistry, University of Tennessee, Knoxville, Tennessee, USA; 5 Department of Pharmacy and Pharmaceutical Sciences, St. Jude Children’s Research Hospital, Memphis, Tennessee, USA; 6 Department of Natural Sciences, University of Michigan-Dearborn, Dearborn, Michigan, USA; Duke University Hospital, Durham, North Carolina, USA

**Keywords:** *Candida*, phosphatidylserine, Cho1, CBR-5884, small-molecule screening

## Abstract

Systemic infections by *Candida* spp. are associated with high mortality rates, partly due to limitations in current antifungals, highlighting the need for novel drugs and drug targets. The fungal phosphatidylserine synthase, Cho1, from *Candida albicans* is a logical antifungal drug target due to its importance in virulence, absence in the host, and conservation among fungal pathogens. Inhibitors of Cho1 could serve as lead compounds for drug development, so we developed a target-based screen for inhibitors of purified Cho1. This enzyme condenses serine and cytidyldiphosphate-diacylglycerol (CDP-DAG) into phosphatidylserine (PS) and releases cytidylmonophosphate (CMP). Accordingly, we developed an *in vitro* nucleotidase-coupled malachite-green-based high throughput assay for purified *C. albicans* Cho1 that monitors CMP production as a proxy for PS synthesis. Over 7,300 molecules curated from repurposing chemical libraries were interrogated in primary and dose-responsivity assays using this platform. The screen had a promising average *Z*’ score of ~0.8, and seven compounds were identified that inhibit Cho1. Three of these, ebselen, LOC14, and CBR-5884, exhibited antifungal effects against *C. albicans* cells, with fungicidal inhibition by ebselen and fungistatic inhibition by LOC14 and CBR-5884. Only CBR-5884 showed evidence of disrupting *in vivo* Cho1 function by inducing phenotypes consistent with the *cho1∆∆* mutant, including a reduction of cellular PS levels. Kinetics curves and computational docking indicate that CBR-5884 competes with serine for binding to Cho1 with a *K*_i_ of 1,550 ± 245.6 nM. Thus, this compound has the potential for development into an antifungal compound.

**IMPORTANCE:**

Fungal phosphatidylserine synthase (Cho1) is a logical antifungal target due to its crucial role in the virulence and viability of various fungal pathogens, and since it is absent in humans, drugs targeted at Cho1 are less likely to cause toxicity in patients. Using fungal Cho1 as a model, there have been two unsuccessful attempts to discover inhibitors for Cho1 homologs in whole-cell screens prior to this study. The compounds identified in these attempts do not act directly on the protein, resulting in the absence of known Cho1 inhibitors. The significance of our research is that we developed a high-throughput target-based assay and identified the first Cho1 inhibitor, CBR-5884, which acts both on the purified protein and its function in the cell. This molecule acts as a competitive inhibitor with a *K*_i_ value of 1,550 ± 245.6 nM and, thus, has the potential for development into a new class of antifungals targeting PS synthase.

## INTRODUCTION

*Candida* species are the most commonly isolated fungal pathogens of humans (1, 2). *Candida* has been associated with mucosal infections, such as vulvovaginal infections in 51% of women or oropharyngeal infections in 27% of HIV + patients, even when on anti-retroviral (ART) therapy (3, 4). In addition, these species are responsible for ~27% of bloodstream infections associated with a central line (1, 2, 5). *Candida* bloodstream infections pose a considerable threat to public health, with a mortality rate of approximately 40% (1, 2, 6, 7). *Candida albicans* is the most commonly isolated species within the *Candida* genus (1, 2, 8). Currently, there are only three classes of antifungal drugs in common use (azoles, polyenes, and echinocandins) for treating systemic *Candida* infections. However, their effectiveness is hindered by rising drug resistance to azoles and echinocandins and the toxicity profile of the polyene amphotericin B (9–13). Therefore, there is a pressing need to develop novel antifungal drugs.

Central in the phospholipid synthetic pathway in fungi is the phosphatidylserine (PS) synthase reaction (CDP-diacylglycerol—serine *O-*phosphatidyltransferase; EC 2.7.8.8) mediated by Cho1. This enzyme has been identified as a potential drug target due to the observations that (i) disruption of Cho1 in *C. albicans* prevents this fungus from causing disease in mouse models of systemic or oral infection (14, 15), and this enzyme is also crucial for the growth of the major fungal pathogen *Cryptococcus neoformans* (16); (ii) Cho1 is not present in mammals, indicating that specific inhibitors targeting this enzyme should not have toxic effects on humans (17), and (iii) *CHO1* is highly conserved across various fungal species, suggesting that inhibitors of this enzyme would have broad spectrum anti-fungal effects (16, 17). Hence, inhibitors of Cho1 would be excellent lead compounds for antifungal drug development.

The fungal Cho1 enzyme was first characterized in the yeast *Saccharomyces cerevisiae*. This included descriptions of cellular localization in the endoplasmic reticulum and mitochondrial-associated membranes (18–20), activity regulation (21–23), and protein purification (24, 25). Cho1 catalyzes the formation of PS from cytidyldiphosphate-diacylglycerol (CDP-DAG) and L-serine, and it belongs to the CDP-alcohol phosphatidyltransferase (CDP-AP) protein family. The CDP-AP proteins employ the highly conserved CDP-alcohol phosphotransferase (CAPT) motif, D-(X)_2_-D-G-(X)_2_-A-R-(X)_2_-N-(X)_5_-G-(X)_2_-L-D-(X)_3_-D, to bind CDP-linked molecules and facilitate the formation of a phosphodiester bond between the CDP-linked molecule and another small alcohol (16, 26–30), specifically CDP-DAG and serine for Cho1. However, it is important to note that the binding pocket for serine, unlike the CAPT motif, is not conserved among CDP-AP proteins. Previous studies have identified and characterized several crucial residues within the CAPT motif and the putative serine-binding site of *C. albicans* Cho1 through alanine scanning mutagenesis (26, 31). Additionally, valuable insights have been provided into the serine-binding pocket from the atomic structure of the PS synthase from the archaean *Methanocaldococcus jannaschii* (32). Differences between the *M. jannaschii* PS synthase and *C. albicans* Cho1 are apparent by the presence of differing numbers of transmembrane domains, oligomeric states, and there are specific residues in *C. albicans* Cho1 that play important roles, but the roles of corresponding residues in *M. jannaschii* PS synthase are unclear (26, 32).

Besides the characterization of the substrate-binding residues, *C. albicans* Cho1 has also been solubilized and purified as a hexameric protein (33), distinct from all the CDP-AP enzymes with solved structures (30, 32, 34–40), which are dimers. The hexameric *C. albicans* Cho1 can be separated into a trimer of stable dimers, indicating the hexamer might be (i) an early oligomer state, since Cho1 was solubilized from the early-to-mid log phase of *C. albicans* or (ii) species-specific (33). Furthermore, purified Cho1 enzyme was optimized for activity and was shown to have a *K*_m_ for CDP-DAG of 72.20 µM with a *V*_max_ of 0.079 nmol/(μg*min) while exhibiting a sigmoidal kinetic curve for its other substrate serine, indicating cooperative binding (33). This sigmoidal kinetic could potentially reconcile the contradicting high and low *K*_m_ values reported previously for *S. cerevisiae* PS synthase (22, 25, 41–43). The mechanism underlying the cooperative binding of serine is currently unknown.

Rational drug design is one way to discover compounds to a drug target protein. This can be achieved through either ligand- or structure-based design methods (44). However, since there is a scarcity of known Cho1 ligands, and the atomic structure of *C. albicans* Cho1 has not yet been solved, employing rational drug design to identify Cho1-specific inhibitors is challenging. On the contrary, small-molecule screening is an alternative way to identify inhibitors to Cho1 independent of structural information. Two whole-cell screens have been carried out to identify Cho1 inhibitors, but neither has been successful (45, 46). One identified the compound SB-224289, but it was discovered that SB-224289 only affects Cho1-associated physiological pathways (45). The other screen identified bleomycin, but this again impacts phospholipid-related physiologies rather than Cho1 itself (46). Thus, to carry out identification of a Cho1 inhibitor, a target-based screen was developed. This approach is enabled by the purification of Cho1 and is favorable because molecules identified will show direct inhibition of the target. Potential issues such as the cellular entry of molecules identified from target-based screening can be resolved later through medicinal chemistry approaches.

Cho1 activity has been measured in crude membrane preps (26, 31, 45, 47) and for the purified form (33, 48) using a radioactive substrate. However, that methodology is not practical for a high throughput screen. Here, we have adapted a non-radioactive assay with an easy setup and colorimetric readout (49), which detects the byproduct cytidine monophosphate (CMP) released from Cho1, to measure its activity in the presence of screening molecules. Using this assay, approximately 7,300 molecules were interrogated in a primary screen from a set of curated repurposing libraries to reveal one compound, CBR-5884, that stood out, as it displayed an inhibitory effect on Cho1 both *in vitro* and in live *C. albicans* cells.

## RESULTS

### A malachite-green-based nucleotidase-coupled assay was used to screen for inhibitors of purified Cho1 protein

**Fig 1:**
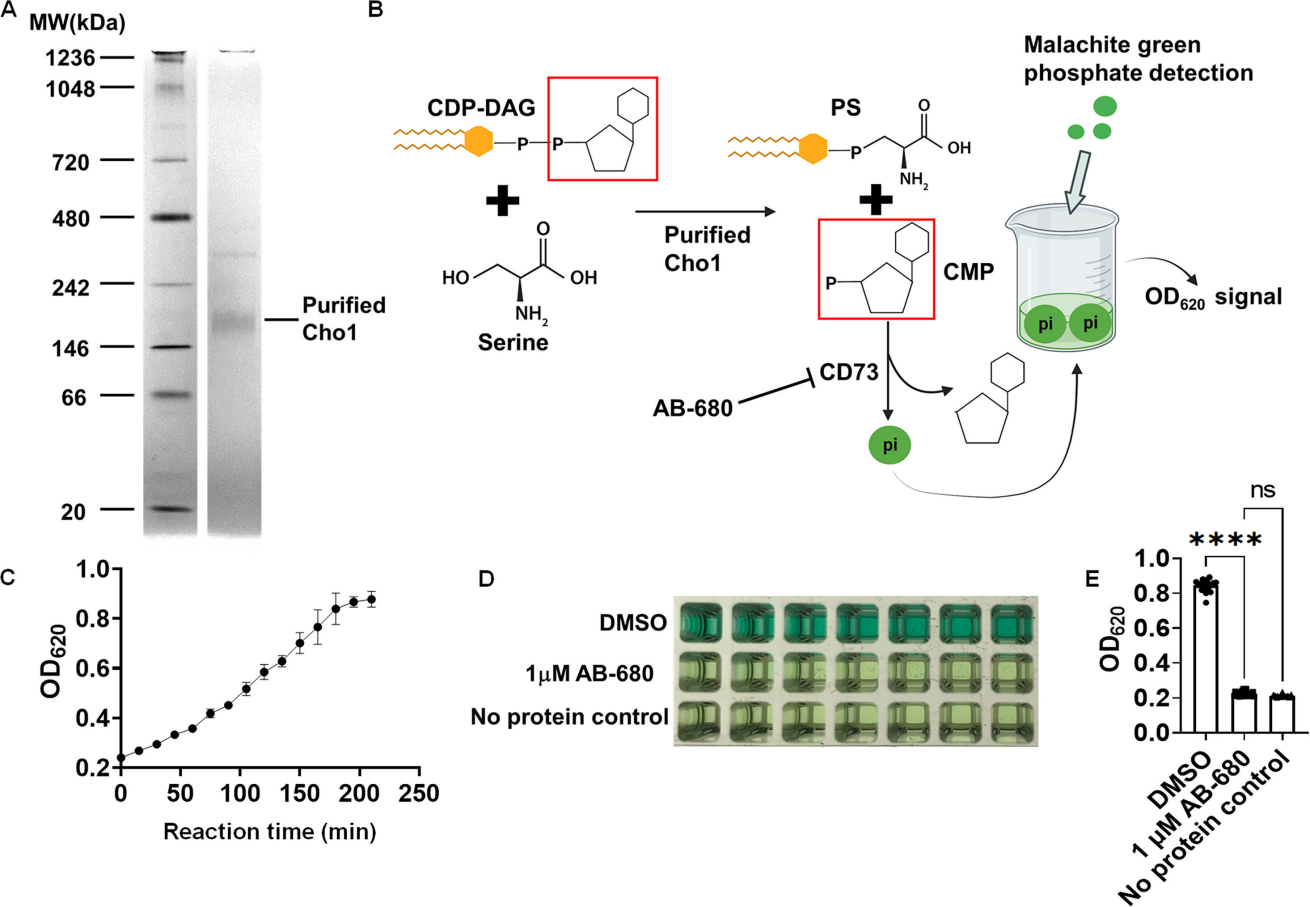
A malachite-green-based nucleotidase-coupled assay measures the activity of purified Cho1 protein. (**A**) Blue native PAGE gel of the purified hexameric tag-free Cho1 protein. Purified Cho1 and protein ladder with known MW are indicated. The gel was stained with Coomassie Blue R-250. (**B**) Schematic representation of the malachite-green-based nucleotidase-couple assay. Cho1 synthesizes PS from CDP-DAG (cytidyldiphosphate-diacylglycerol) and serine. This releases PS and CMP (cytidylmonophosphate). The phosphate from CMP is cleaved by the nucleotidase CD73 to release inorganic phosphate, which can be bound by the malachite green reagent and measured colorimetrically at OD_620_. AB-680 is a potent inhibitor of CD73 and can, thus, inhibit the reaction. (**C**) OD_620_ signal from the malachite green reagent that was added to the reaction (shown in **B**) at different time points after the reaction started. Reactions were set up with the same conditions and stopped by adding malachite green at the time indicated. The dots represent the mean of four biological replicates, and the error bars are ±standard deviation (S.D.) values. (**D**) Inhibition of the nucleotidase-coupled assay by AB-680 is shown for a series of replicates in 384-well format and (**E**) is quantified for a total of 21 replicates. Statistics were conducted using one-way ANOVA using Tukey’s multiple comparisons test (ns, not significant; ****P < 0.0001).

An expression cassette plasmid carrying the strong, constitutive promoter for translational elongation factor 1 (*P_TEF1_*) fused upstream of the 8x-histidine tagged *C. albicans CHO1* gene was integrated into the genome at the *TEF1* locus to ensure a strong and stable expression level (50). Then, Cho1 was solubilized and purified from the microsomal fraction of *Candida albicans* as described in the Materials and Methods. A blue native PAGE indicated that the Cho1 protein was purified to relative homogeneity as a hexameric form of ~180 kDa (Fig. 1A), consistent with findings in reference (33). The purified Cho1 was used for small molecule screening.

A malachite-green-based nucleotidase-coupled assay was used to measure the PS synthesis activity of Cho1 (Fig. 1B). Cho1 catalyzes the production of PS and cytidylmonophosphate (CMP) from CDP-DAG and serine, where CMP can then be recognized and cleaved by the nucleotidase CD73 to release inorganic phosphate, which is subsequently detected by malachite green. To test whether this malachite-green-based assay can reflect Cho1 activity, a time-course test was performed with a fixed amount of purified Cho1 in the presence of substrates (Fig. 1C). The OD_620_ signal increased as the reaction proceeded until it plateaued at 200 min, indicating that the assay is suitably dynamic to probe Cho1 activity. Based on this data, a fixed time of 180 min was chosen for the screen.

As no effective inhibitors of Cho1 have been identified to date, a highly potent and selective inhibitor of the second step of the assay (nucleotidase CD73) was examined as a positive control (51). This compound, AB-680, exhibited an IC_50_ value of 1.82 nM in the malachite-green assay (Fig. S1A). A concentration of 1 µM AB-680 was used in the screen and was compared to DMSO and no protein wells, which served as negative controls. Reactions with AB-680 showed similar color as the no protein control in the 384-well plate (Fig. 1D), and the measured OD_620_ signal was fourfold less in the presence of the AB-680 (Fig. 1E). Thus, AB-680 can be used as a positive control for identifying inhibitors in the primary screen. In order to eliminate false positives that are actually inhibiting the nucleotidase CD73, a counter-screening method was developed. In this method, Cho1 was substituted with CMP, while CD73 remained included to identify any compounds that directly inhibit the nucleotidase instead of Cho1.

### Seven Cho1-specific inhibitors were identified from the high-throughput small molecule screen

**Fig 2:**
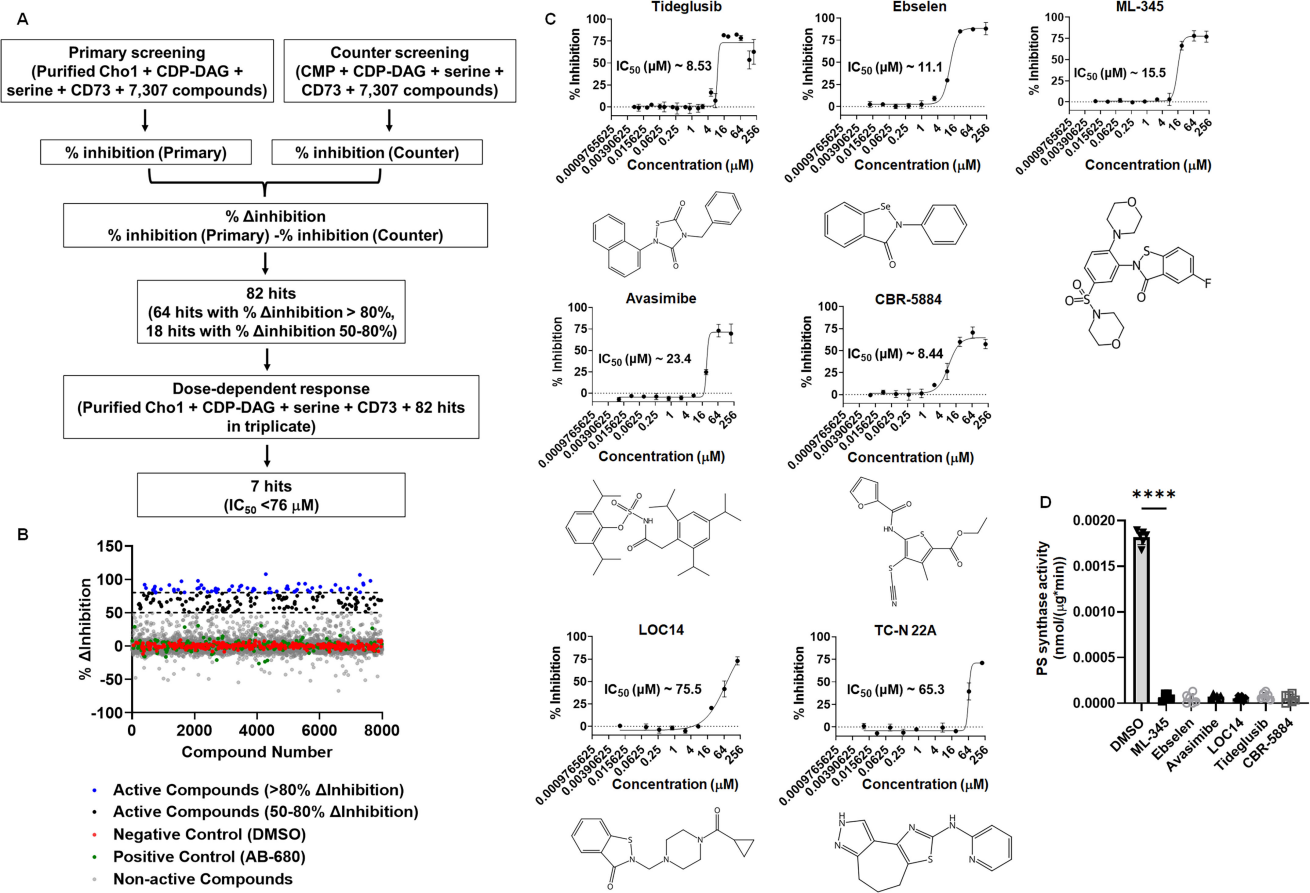
Seven Cho1-specific inhibitors were identified from the high-throughput malachite green screen. (**A**) Flowchart for the primary and counter screen and the calculation of % Δinhibition. (**B**) The dot plot of % Δinhibition for all the compounds, including controls, used in the screen. Reaction with DMSO and AB-680 was used as negative and positive controls, respectively. The two dotted lines from the *Y*-axis indicate 80% and 50% Δinhibition, respectively. (**C**) Dose-response curve and structure of the seven non-pan-assay interference (non-PAINS) compounds identified from the screen. The dots represent the mean of three replicates, and the error bars are ±standard deviation (S.D.) values. Best-fit IC_50_ values (in μM) were shown in each graph. (**D**) The PS synthase activity of purified Cho1 was measured by L-[^3^H]-serine incorporation into PS in the presence of different inhibitors at 100 µM or equivalent DMSO and are presented as nmol/(μg protein*min). Statistics were conducted using one-way ANOVA and Dunnett’s T3 multiple comparisons test (****P > 0.0001). The activities were measured in duplicate with a total of six biological replicates as indicated. The bars represent the mean and the error bars are ±S.D. values.

The screen interrogated 7,307 molecules from three curated repurposing libraries in the primary screen and counter screen (which were run concurrently) at a final concentration of 100 µM each (Fig. 2A). The primary screen had an average *Z*′ score of ~0.8 (Fig. S1B), indicating a good signal-to-noise ratio (52). To prioritize hit compounds and eliminate false positives, % Δinhibition (Fig. 2B) was calculated by subtracting the % inhibition from the counter screen (Fig. S1D) from that of the primary screen (Fig. S1C) for each molecule. All compounds from the >80% Δinhibition population and selected molecules with limited structural liabilities from the 50%–80% Δinhibition population (for a total of 82 molecules) were advanced for dose-response assessment using the same screening platform, which once again yielded results of high quality (*Z′* of 0.83; Fig. S1E). Compounds exerting dose-dependent activity were then further triaged by manual inspection to exclude pan assay interference compounds (PAINS), which tend to react nonspecifically with numerous biological targets (53). Finally, seven molecules exerting IC_50_ values ≤76 µM were identified, of which CBR-5884, ML-345, ebselen, and tideglusib were the most potent, possessing IC_50_ ≤20 µM (Fig. 2C). These molecules, in addition to avasimibe and LOC14, were selected for further investigation. The TC-N 22A molecule was not easily available and was not pursued.

To further validate the inhibitory effects of the six molecules on Cho1, a radioactive PS synthase assay was conducted on Cho1 in the presence of these compounds. Unlike the malachite-green-based assay, the radioactive PS synthase assay directly measures the incorporation of L-[^3^H]-serine into PS in the lipid phase (26, 33, 47). The radioactive PS synthase assay was performed on purified Cho1 in the presence of the six compounds at 100 µM, and all six molecules were shown to totally inhibit Cho1 (Fig. 2D), consistent with the screening results.

### Ebselen, LOC14, and CBR-5884 showed inhibitory effects on *C. albicans* cells

**Fig 3:**
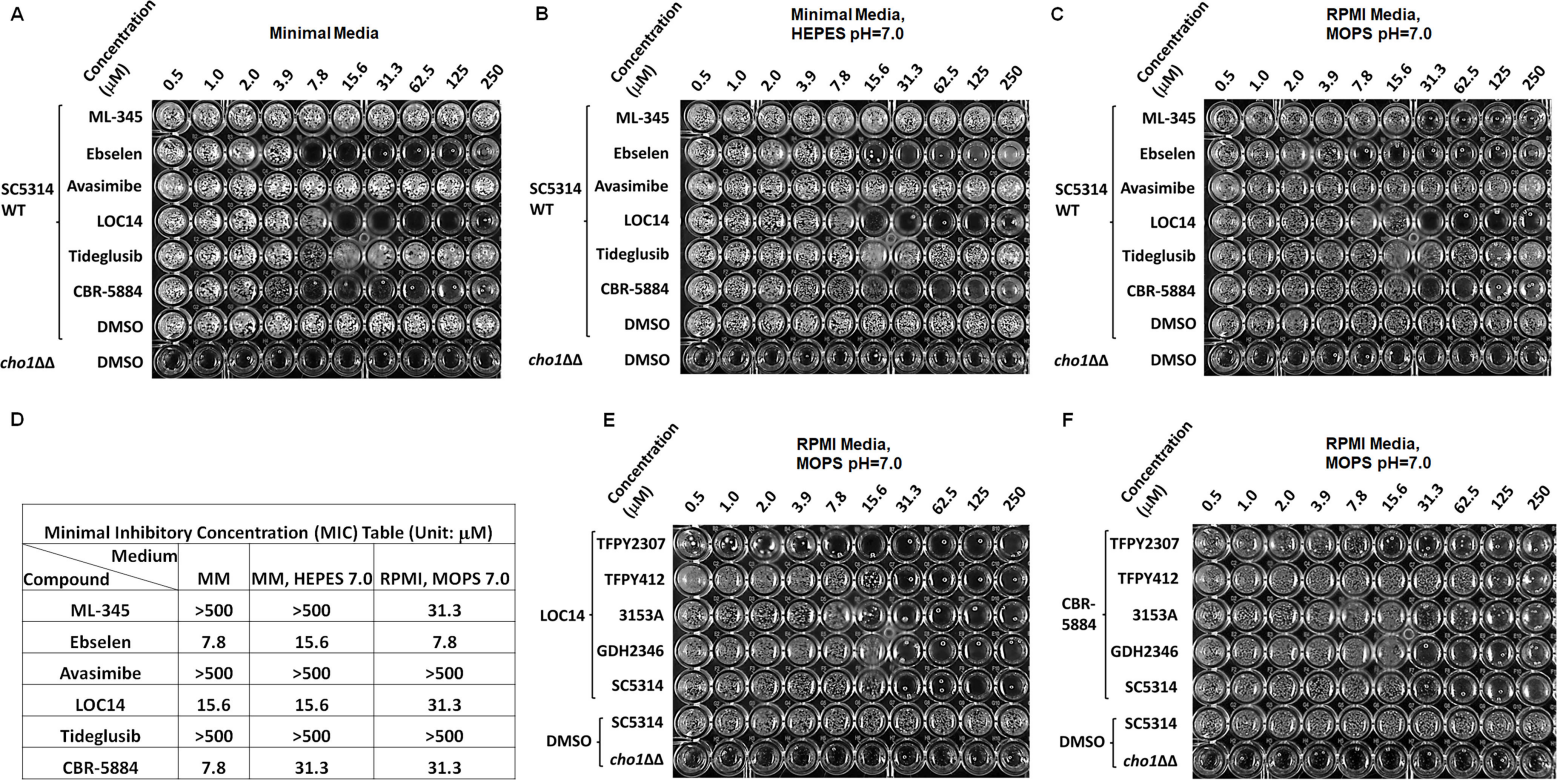
Ebselen, LOC14, and CBR-5884 inhibited cell growth of *C. albicans* in different media. (**A–C**) The MIC was measured for each compound in three different media for 48 h: (**A**) minimal medium, (**B**) minimal medium buffered with 50 mM HEPES (pH 7.0), and (**C**) RPMI MOPS (pH 7.0). These tests were conducted, by standard CLSI MIC broth microdilution protocols, against the wild-type SC5314 strain, using DMSO as a negative control, and against the *cho1*∆∆ mutant with DMSO serving as a positive control. (**D**) A summary table of all MIC values from (**A–C**). (**E and F**) Multiple wild-type *C. albicans* isolates were tested with different concentrations of (**E**) LOC14 and (**F**) CBR-5884. All concentrations indicated are micromolar (μM).

We then tested the effects of the six compounds on live cells by determining the minimal inhibitory concentrations (MICs) of these compounds. Following the standard Clinical & Lab Standards Institute (CLSI) MIC broth microdilution methods, wild-type *C. albicans* strain SC5314 was grown with avasimibe, CBR-5884, tideglusib, ebselen, ML-345, and LOC14 in RPMI MOPS medium (pH 7.0) or alternatively in minimal medium or minimal medium with HEPES (pH 7.0) at 37°C for 48 h, along with a *cho1*ΔΔ strain as a control (Fig. 3A through C). The concentration of the compounds was varied from 0.5 to 250 µM. Avasimibe, tideglusib, and CBR-5884 precipitated at high concentrations, indicating a low solubility (Fig. S2A). Among all compounds, avasimibe and tideglusib did not affect cell growth in all three different media, even at 250 µM, indicating they do not have an inhibitory effect on *C. albicans* cells under our assay conditions (Fig. 3A through C). ML-345 only inhibited cell growth in RPMI MOPS medium with an MIC of 31.3 µM and had no effect on cells grown in the minimal media. Ebselen, LOC14, and CBR-5884, on the contrary, stopped cell growth at different concentrations in all three media, with MICs ranging between 7.8 and 31.3 μM (Fig. 3A through D). This is consistent with the radioactive PS synthase assay done on the crude membrane containing Cho1, in which only ebselen, CBR-5884, and LOC14 decreased PS production in the native crude membranes (Fig. S3). Specific MIC values are summarized in Fig. 3D for all the compounds.

Furthermore, there are no current reports on the antifungal effects of LOC14 and CBR-5884. To rule out the possibility that their inhibition is strain-specific, we treated four additional *C. albicans* wildtype strains—GDH2346 (54), 3153A (55), and two clinical isolates TFPY412 and TFPY2307—with LOC14 and CBR-5884. Tests were done in RPMI MOPS (pH 7.0) medium at 37°C for 48 h following the CLSI MIC broth microdilution protocol, and a *cho1ΔΔ* strain was included as a control (Fig. 3E and F). As shown, all strains were inhibited by LOC14 and CBR-5884 at varying concentrations, indicating their inhibition is not specific to certain strains.

### The inhibitory effect of CBR-5884 can be rescued by ethanolamine supplementation

**Fig 4:**
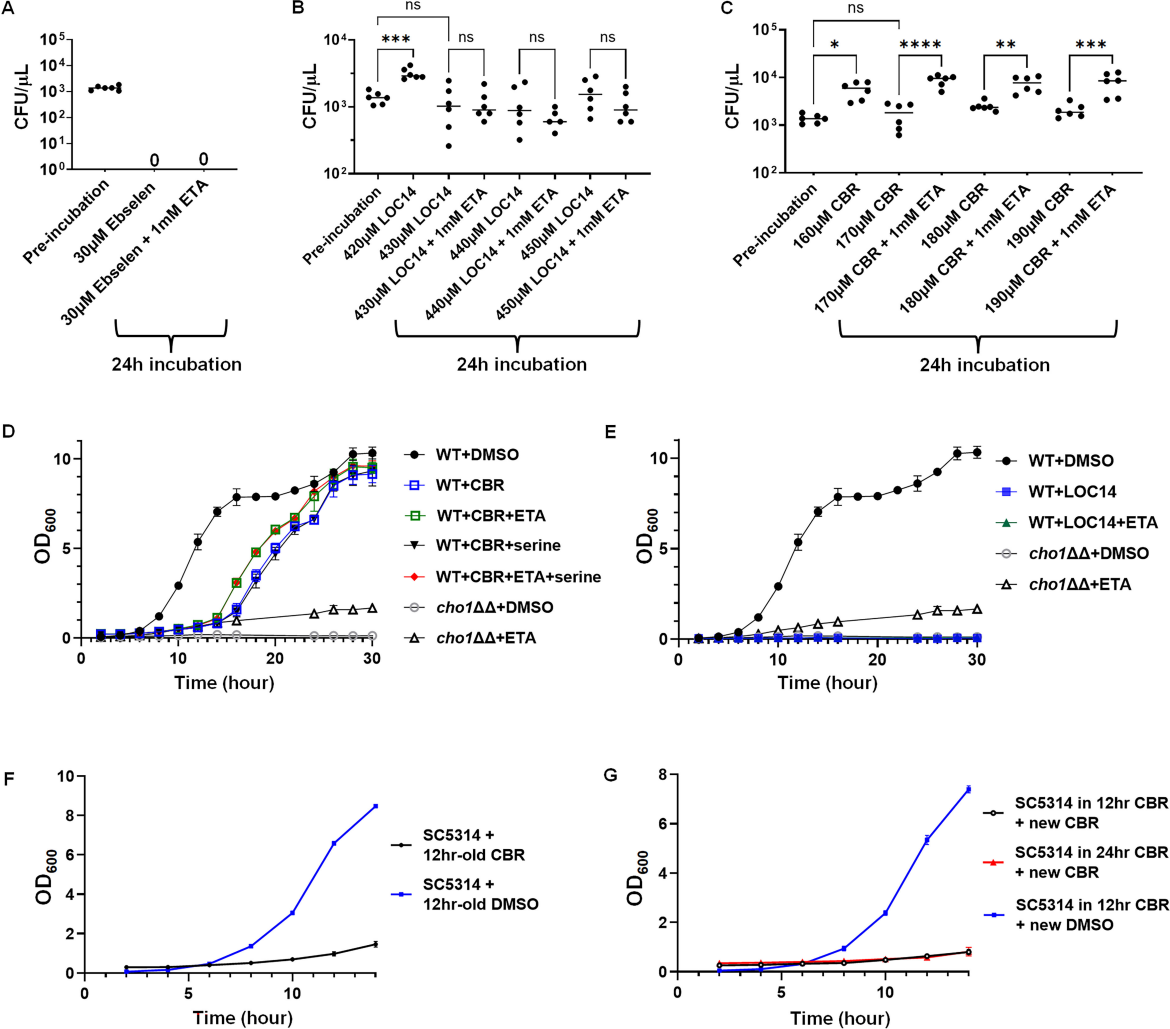
Ethanolamine supplementation can mitigate the inhibitory effects of CBR-5884. (**A–C**) Colony-forming units from cells treated with different concentrations of (**A**) ebselen, (**B**) LOC14, and (**C**) CBR-5884, ± 1 mM ethanolamine (ETA), for 24 h compared to pre-incubation. Statistics were conducted using one-way ANOVA and Tukey’s multiple comparisons test (ns, not significant; *P* > 0.05; *, 0.05 > *P* > 0.01, **, 0.01 > *P* > 0.001). Six biological replicates were tested in each treatment. (**D**) Growth curves of wild-type *C. albicans* in the presence of 170 µM CBR-5884 or equivalent DMSO, with the addition of 1 mM ethanolamine (ETA) or 5 mM serine or both, from 0 to 30 h. The *cho1*ΔΔ strain was also included as a control. (**E**) Growth curves of wild-type *C. albicans* in the presence of 430 µM LOC-5884 or equivalent DMSO, with the addition of 1 mM ethanolamine (ETA), from 0 to 30 h. (**F**) Minimal medium supplemented with 170 µM CBR-5884 or equivalent DMSO was agitated for 12 h before the introduction of wild-type *C. albicans* SC5314 strain, and growth curves were recorded from 0 to 14 h. (**G**) Wild-type *C. albicans* SC5314 strain was grown in 170 µM CBR-5884 for 12 and 24 h before being diluted in fresh minimal medium supplemented with 170 µM CBR-5884 or equivalent DMSO. The growth curves were recorded from 0 to 14 h. The dots in (**D–G**) represent the mean values of six biological replicates, and the error bars are ± standard deviation (S.D.) values.

The antifungal effects of ebselen, LOC14 ,and CBR-5884 on live cells were further evaluated by plate assays and growth curves. First, we tried to determine if the inhibitory effects of these compounds were fungistatic or fungicidal. It should be highlighted that in contrast to the MIC assays, the plate assays and growth curve analyses necessitated a greater initial inoculum (OD_600_ of 0.05 as opposed to 0.00004) for detection. Additionally, a reduced incubation temperature of 30°C instead of 37°C was required to inhibit the hyphal formation, which, in turn, required the adjustment of compound concentrations used. For this, wild-type *C. albicans* strain SC5314 was grown with varying concentrations of ebselen, LOC14, and CBR-5884 in minimal media at 30°C for 24 h, along with a *cho1*ΔΔ strain as a control. Under this condition, ebselen suppressed cell growth at concentrations from 15.9 to 31.8 µM, LOC14 showed inhibition within the range of 250–500 µM, and CBR-5884 was effective between 125 and 250 µM, as reflected by both the decreased growth in the 96-well plates and reduced cell density in the microscope images in the higher concentrations (Fig. S4). To further determine the optimized inhibitory concentrations and whether the inhibition is fungistatic or fungicidal, wild-type *C. abicans* SC5314 was then incubated with ebselen, LOC14, and CBR-5884 in a dosage series with 10 µM increments within the inhibition range determined above, and colony forming units (CFUs) were counted after 24 h incubation. Ebselen showed no colonies after incubation at 30 µM, which is consistent with a fungicidal effect (Fig. 4A). In contrast, cells incubated with 430 µM LOC14 and 170 µM CBR-5884 exhibited similar CFUs as the pre-incubation, indicative of a fungistatic effect (Fig. 4B and C).

Next, we determined if the impacts of ebselen, LOC14, and CBR-5884 on cells were consistent with perturbed Cho1 function. Cho1 synthesizes PS via the *de novo* pathway, and PS is the precursor for making the essential phospholipid phosphatidylethanolamine (PE). When Cho1 is inhibited, growth can only resume, albeit more slowly, if the organism is able to make PE from ethanolamine acquired from the medium via the salvage (Kennedy) pathway (14, 26). Thus, ethanolamine-dependent growth in minimal media is a characteristic phenotype of PS synthesis loss. With this, the growth inhibition assay was repeated at the optimized drug concentrations in minimal medium supplemented with 1 mM ethanolamine, and an increase in the CFUs in the presence of ethanolamine indicates an inhibition on Cho1 function. Although ethanolamine did not rescue cells with ebselen or LOC14, cells treated with the ≥170 µM CBR-5884 generated significantly higher CFUs upon the addition of ethanolamine (Fig. 4A through C). This result suggests that the inhibitory effect of ebselen and LOC14 on the cells is not solely caused by Cho1 inhibition, while CBR-5884 is more directly targeting Cho1.

To gain insight into the dynamic inhibitory properties of these molecules, growth curves were determined with wild-type cells grown in the minimal media ± ethanolamine, in the presence of CBR-5884 or LOC14. Ebselen was not pursued in the growth curve assay due to its fungicidal effect. The *cho1*ΔΔ strain was used as a control for loss of Cho1 activity. Given that CBR-5884 is a selective inhibitor of phosphoglycerate dehydrogenase that is involved in *de novo* serine synthesis in cancer cells (56), and to make sure that the growth perturbation is not due to serine starvation, 5 mM serine was also added to the media ± ethanolamine. As shown in Fig. 4D, cells grown in media + ethanolamine and +ethanolamine/serine grew similarly, while those grown in minimal media and minimal media + serine had similarly reduced growth. This suggests that serine did not help the cells recover from the inhibition from CBR-5884, which likely indicates (i) CBR-5884 does not target *C.albicans* phosphoglycerate dehydrogenase or (ii) the CBR-5884 inhibition of *C. albicans* phosphoglycerate dehydrogenase is not the major cause of diminished growth. Cells with ethanolamine supplementation grew better than those in minimal media alone, especially from 12 to 24 h, consistent with Fig. 4C. Growth rates during log phase (Fig. S2B) and lag phase duration (Fig. S2C) were estimated from the growth curves, and they showed that the addition of ethanolamine significantly increased the growth rate and decreased the lag time. All of these results again support the hypothesis that CBR-5884 targets Cho1 *in vivo*.

However, it was observed that the impact of CBR-5884 on cell growth was only temporary, with a delay lasting 12 h. After this period, cells treated with CBR-5884 exhibited rapid growth, eventually matching the growth rate of the DMSO control group following a 24 h incubation period (Fig. 4D). This suggests that either (i) CBR-5884 lost its effect after 12 h of agitation or (ii) that *C. albicans* cells acquire resistance to the compound within this timeframe. To disentangle these two possibilities, experiments were conducted where both CBR-5884 and DMSO were subjected to 12 h of agitation at 30°C before being introduced to live cells. The growth of these cells was monitored over the next 14 h, with an OD_600_ measurement every 2 h, to assess the persistence of CBR-5884’s efficacy (Fig. 4F); in the meantime, cells that had been grown in 170 µM CBR-5884 for 12 and 24 h were transferred into fresh media containing new CBR-5884, which was allowed to grow for another 14 h, to probe the rising of resistance (Fig. 4G). In both experimental setups, cells grew slowly in the presence of CBR-5884 compound compared to DMSO control. This suggests that (i) CBR-5884 maintains its stability and effectiveness under agitation for at least 12 h, and (ii) the breakout growth in Fig. 4D is not due to the rise of resistance to CBR-5884. One possible explanation could be that *C.albicans* cells metabolize the CBR-5884 compound, making it inactive after 12 h and resulting in a surge in growth.

Furthermore, LOC14 was also subjected to growth curve determination but no cells grew, in the presence or absence of ethanolamine (Fig. 4E). This corroborates with Fig. 4B that the inhibition by LOC14 is not acting solely on Cho1. Since the goal was a Cho1-specific inhibitor, ebselen and LOC14 were not pursued further.

### CBR-5884 interferes with PS synthesis *in vivo*

**Fig 5:**
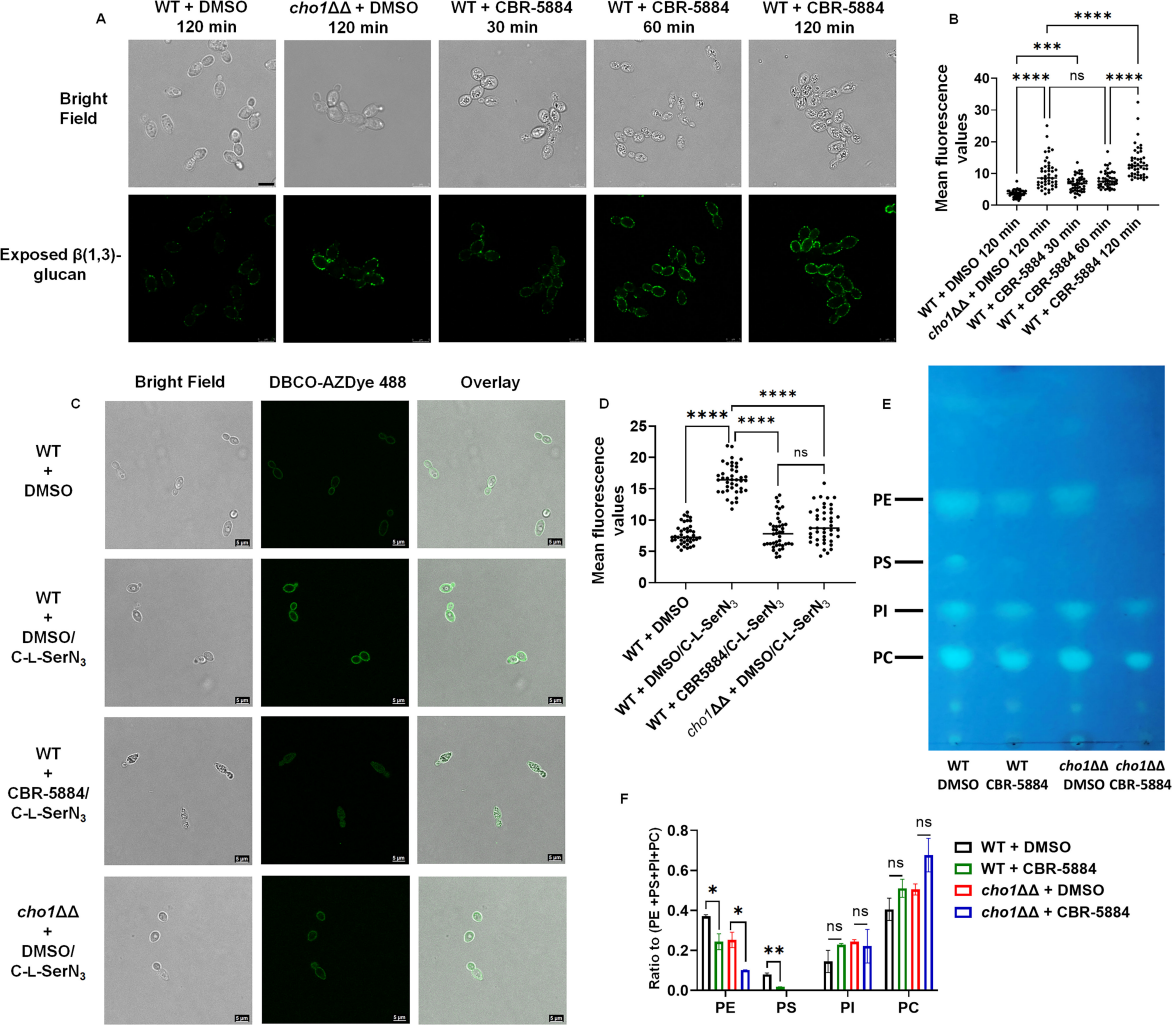
CBR-5884 interferes with *in vivo* PS synthesis. (**A**) CBR-5884 induces cell wall β(1,3)-glucan exposure. Exposure of cell wall β(1,3)-glucan by wild-type *C. albicans* treated with 170 µM CBR-5884 or equivalent DMSO control was measured at the indicated time points. Exposed β(1,3)-glucan is shown as green fluorescence, and the corresponding bright field images are shown. (**B**) Quantification of the mean fluorescence from (**A**). Forty-six cells from at least 10 fields of view were used for the quantification for each condition. Statistics were conducted using one-way ANOVA and Tukey’s multiple comparisons test (ns, not significant, *P* > 0.05; ****, 0.0001 > *P*). (**C**) CBR-5884 interferes with the incorporation of C-l-SerN_3_ probe into the cell membrane. A final concentration of 1.5 mM C-l-SerN_3_ was added to wild-type *C. albicans* and *cho1*ΔΔ cells grown with and without 170 µM CBR-5884. The cells were then stained with the DBCO-AZ Dye 488 to allow click-tagging, followed by microscopy. Corresponding brightfield and overlay images were also shown. Wild-type *C. albicans* grown without the C-l-SerN_3_ probe was included as a control for background fluorescence. (**D**) Quantification of the mean fluorescence from (**C**). Forty cells from at least 10 fields of view for each condition were used for the quantification. Statistics were conducted using one-way ANOVA and Tukey’s multiple comparisons test (ns, not significant, *P* > 0.05; ****, 0.0001 > *P*). (**E**) Thin-layer chromatography (TLC) plate of phospholipids extracted from wild-type and *cho1*ΔΔ *C. albicans* treated with 170 µM CBR-5884 or equivalent DMSO. The positions of PS (phosphatidylserine), PE (phosphatidylethanolamine), PI (phosphatidylinositol), and PC (phosphatidylcholine) are indicated based on standards. (**F**) The ratio of the phospholipid to total (PE + PS + PI + PC) phospholipids for strains in (**E**). The quantification was done in ImageJ software from two TLC plates. Statistics were conducted using unpaired two-tailed *t* test (ns, not significant, *P* > 0.05; *, 0.05 > *P* > 0.01, **, 0.01 > *P* > 0.001).

It has been determined previously that deletion of Cho1 leads to increased exposure (unmasking) of cell wall β(1-3)-glucan, rendering cells more prone to be targeted by the immune system (14, 57). Here, we tested whether CBR-5884 could induce unmasking. Wild-type *C. albicans* cells were grown in rich medium (YPD) supplemented with DMSO or CBR-5884 for 30, 60, and 120 min, along with the *cho1*ΔΔ mutant control, and exposed β(1-3)-glucan was stained and visualized through confocal microscopy. The *cho1*ΔΔ strain exhibited increased unmasked foci, compared to the WT strain in DMSO, consistent with previous findings that disruption of Cho1 leads to increased unmasking (Fig. 5A) (57). Similarly, CBR-5884 treatment showed increased unmasking after 30 min incubations, and the unmasking became more obvious at 60- and 120-min treatment. The mean fluorescence values confirmed that a 30-min CBR-5884 treatment is sufficient to induce significantly increased unmasking compared to wildtype *C. albicans*, and 120-min treatment could induce more unmasking than the *cho1*ΔΔ mutant (Fig. 5B). This could be because CBR-5884 inhibition causes a sudden loss of Cho1 function that the cells have not yet adjusted to, whereas a *cho1∆∆* mutant has adjusted its metabolism to the loss of PS. Alternatively, it may indicate an off-target effect. It is also interesting to note that there are dark structures in the cells under bright field microscopy when treated with CBR-5884, which are absent in wild-type or *cho1*ΔΔ strains without CBR-5884. We currently cannot explain the identity or formation of these structures but speculate that they represent fragmented vacuoles that serve in detoxification (58).

In order to more directly measure the impact of CBR-5884 on PS synthesis *in vivo*, an assay for fluorescence-based labeling of PS via biorthogonal tagging using a clickable serine probe was utilized. This probe consists of a serine analog carrying an azide tag for click chemistry, C-l-SerN_3_. This probe has been demonstrated to be incorporated into live cell membranes by infiltrating lipid metabolism to produce azide-tagged PS analogs that can be post-labeled by click-tagging with fluorophores to localized/quantify PS in cells (59, 60). Wild-type *C. albicans* was grown in YPD to early log phase before C-l-SerN_3_ was added, along with CBR-5884 or DMSO (Fig. 5C). The *cho1*ΔΔ strain and no probe controls were also included for background fluorescence, and mean fluorescence of each group was quantified (Fig. 5D). In the absence of CBR-5884, C-l-SerN_3_ labels the cell membrane of wild-type *C. albicans* with stronger fluorescence compared to no probe or *cho1*ΔΔ strains (Fig. 5C and D), indicating C-l-SerN_3_ was converted into PS as previously described (59). However, in the presence of CBR-5884, the fluorescence is significantly diminished on the periphery of the cell (Fig. 5C and D). This indicates that CBR-5884 inhibits *in vivo* PS production.

Finally, a direct biochemical test for PS levels was performed by thin-layer chromatography (TLC) in cells treated with CBR-5884. Wild-type *C. albicans* and *cho1*ΔΔ strains were grown in the presence and absence of CBR-5884, and the four major phospholipid species are shown in Fig. 5E and quantified in Fig. 5F. Consistently, the relative PS level in the wild-type *C. albicans* strain treated with CBR-5884 significantly dropped compared to the DMSO treatment (Fig. 5E and F), indicating that CBR-5884 interferes with PS production. Interestingly, the relative PE levels also significantly decreased in strains treated with CBR-5884, especially in the *cho1*ΔΔ strain without PS as a precursor (Fig. 5E and F). This potentially indicates that CBR-5884 may independently impact PE production.

### CBR-5884 acts as a competitive inhibitor that occupies the serine-binding site of Cho1

**Fig 6:**
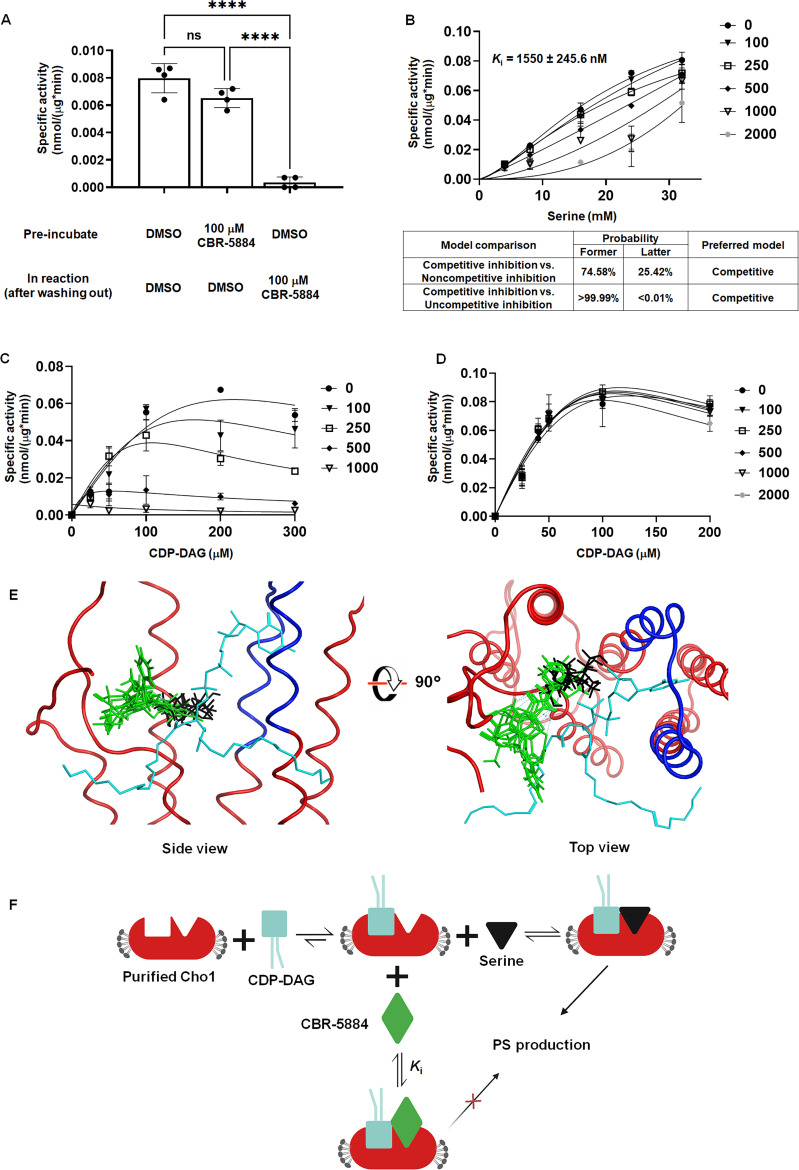
CBR-5884 may function as a competitive inhibitor occupying the serine-binding site of Cho1. (**A**) Purified Cho1 was pre-incubated with DMSO or 100 µM CBR-5884 for 2 h before being washed out and tested against DMSO or 100 µM CBR-5884. Four specific activities were measured for each condition, and statistics were conducted using one-way ANOVA and Tukey’s multiple comparisons test (ns, not significant, ****, 0.0001 > *P*). (**B**) Kinetic curves for serine in the presence of CBR-5884. CDP-DAG was kept constant at 200 µM (4.8 mol %, *K*_m_ = 36.66 ± 11.10 µM), and the specific activities of purified hexameric Cho1 were plotted against various serine concentrations (4, 8, 16, 24, 32 mM) in the presence of 0, 100, 250, 500, 1,000, 2,000 nM CBR-5884. The curves best fit for competitive inhibition with a *K*_i_ value of 1,550 ± 245.6 nM. The dots in all curves represent the mean values of two biological replicates, and the error bars are ±standard deviation (S.D.) values. (**C**) Kinetic curves for CDP-DAG in the presence of CBR-5884. Serine was kept constant at 20 mM (*K*_half_ = 17.08 ± 4.072 mM), and the specific activities of purified hexameric Cho1 were plotted against various CDP-DAG concentrations (25, 50, 100, 200, 300 µM, 4.2–5.0 mol %) in the presence of 0, 100, 250, 500, and 1,000 nM CBR-5884. (**D**) Kinetic curves for CDP-DAG in the presence of CBR-5884 with 32 mM serine (*K*_half_ = 17.08 ± 4.072 mM). Specific activities of purified hexameric Cho1 were plotted against various CDP-DAG concentrations in the presence of 0, 100, 250, 500, 1,000, and 2,000 nM CBR-5884. (**E**) Computational docking of serine and CBR-5884 into the Cho1 AlphaFold structure. The top five poses of CBR-5884 (green) and serine (black) are shown within the active site of Cho1. CDP-DAG is shown as cyan and the conserved CAPT motif of Cho1 is highlighted in dark blue. (**F**) A model for the inhibition mechanism of CBR-5884 to Cho1. Cho1 follows a sequential bi-bi reaction, in which it has to bind CDP-DAG prior to serine for catalysis. In the presence of CBR-5884, CDP-DAG-bound Cho1 could either bind serine for a reaction or CBR-5884 for no reaction.

To determine whether CBR-5884 is an irreversible inhibitor, which forms covalent bonds with Cho1, the compound was pre-incubated with purified Cho1 protein for 2 h, followed by a buffer exchange and washout before the reaction (Fig. 6A). The absence of inhibition after preincubation and washout suggests that CBR-5884 does not inhibit the enzyme covalently. To investigate the molecular mechanism by which CBR-5884 inhibits Cho1, purified Cho1 specific activity was assayed with varying concentrations of serine and CDP-DAG in the presence of CBR-5884. Serine was varied from 4 to 32 mM, CDP-DAG was kept at 200 µM, and several concentrations of CBR-5884 were tested (Fig. 6B). The pattern of inhibition more closely fits with competitive inhibition and a low *K*_i_ value of 1,550 ± 245.6 nM. Next, serine was held at a sub-saturating concentration of 20 mM or a saturating concentration of 32 mM, and CDP-DAG was varied from 25 to 300 µM. At the lower serine concentration, the inhibition of CBR-5884 on Cho1 activity was observed (Fig. 6C), but the inhibition was overcome under saturating serine concentrations (Fig. 6D). These results suggest that CBR-5884 inhibits Cho1 by competing for serine and can be outcompeted with a high serine concentration. It is interesting that CDP-DAG inhibits Cho1 activity at high concentrations, especially in the presence of CBR-5884 (Fig. 6C). This substrate inhibition from CDP-DAG has been reported previously (22, 61).

To gather more insight for the serine competition, we computationally docked CBR-5884 into the active site of *C. albicans* Cho1. Since fungal PS synthases follow the ordered sequential bi-bi reaction mechanism where Cho1 binds CDP-DAG before serine for catalysis (25), we first generated a predicted CDP-DAG-bound *C. albicans* Cho1 structure by superposing the *C. albicans* Cho1 AlphaFold model on the CDP-DAG-bound PS synthase from *Methanocaldococcus jannaschii* (PDB: 7B1L) (Fig. S5A) (32). The CDP-alcohol phosphotransferase (CAPT)-binding motif, which is known to bind CDP-DAG, is conserved and aligned between the *C. albicans* Cho1 and *M. jannaschii* PS synthase structures, so we hypothesized that the CDP-DAG from *M. jannaschii* PS synthase interacts with *C. albicans* Cho1 in a very similarly manner and, thus, is incorporated in the *C. albicans* Cho1 model for docking. Next, we simulated and combined all the possible active site pockets from the CDP-DAG-bound *C. albicans* Cho1 and docked CBR-5884 and L-serine into these possible sites (Fig. S5B). A total of 20,000 initial poses of CBR-5884 and serine were generated and then refined to the five top poses with the highest docking scores (Fig. 6E). All five CBR-5884 poses have overlap with the serine poses, with a higher docking score of −11.48 ± 0.12 kcal/mol compared to the serine docking score of −6.72 ± 0.08 kcal/mol. This corroborates the low *K*_i_ value and suggests that CBR-5884 likely competes with serine to occupy the serine binding pocket in Cho1. From this, we generated a working model for CBR-5884 inhibition (Fig. 6F). Following the first step in the sequential bi-bi reaction where Cho1 binds CDP-DAG, either CBR-5884 or serine can dock into the active site. The catalysis will occur if serine enters and will not if CBR-5884 occupies the site. Since CBR-5884 is favored by Cho1, serine can only outcompete CBR-5884 at a high concentration.

## DISCUSSION

Here, we adapted a malachite-green-based nucleotidase-coupled assay to screen for and identify inhibitors targeting *C. albicans* Cho1 (49). Since the amount of phosphate released is directly proportional to the CMP, and thus PS, produced in the reaction, this method can be used to measure Cho1 activity in real time (Fig. 1C). It is worth mentioning that besides CMP, the nucleotidase CD73 is known to cleave AMP to release phosphate in various studies (62–64), and given that this assay is suitable for 384-well plates or even 1536-well plates, it may potentially be applied to any enzyme producing AMP/CMP.

Ebselen, LOC14, and CBR-5884 stood out among seven Cho1-specific inhibitors due to their inhibitory effects on the *C. albicans* cell growth (Fig. 3). Ebselen is an organo-selenium compound originally developed as a glutathione peroxidase mimic that acts on the cholesterol ester hydroperoxides and phospholipid hydroperoxides (65), and it has garnered significant attention in recent years due to its diverse therapeutic applications and anti-inflammatory, antioxidant, and anticancer activity (66–71). In addition, ebselen has exhibited notable *in vitro* and *in vivo* antifungal activity against a range of fungal pathogens, including *Candida* spp., *Fusarium* spp., *Aspergillus fumigatus,* and *Cryptococcus neoformans* (72–74). Studies have shown that ebselen effectively inhibits fungal growth by targeting many key enzymes (75–80). Here, we have demonstrated again that ebselen inhibits wild-type *C. albicans* growth (Fig. 3 and 4). The inhibition of purified Cho1 by ebselen is potent, with an IC_50_ of 11.1 µM (Fig. 2C), but the inhibition on Cho1 is very likely not the main cause for ebselen’s inhibition of *C. albicans* growth since ethanolamine cannot bypass the drug (Fig. 4). Currently, we do not know the mechanism of ebselen’s inhibition on Cho1, but given the tendency for ebselen to interact with cysteine residues (81), we hypothesize that ebselen might interact with residue C182, located in the putative serine-binding site of Cho1 (26), thus disrupting activity. Ebselen’s promiscuous nature limits its clinical applicability.

Conversely, the antifungal effects of LOC14 and CBR-5884 have not been studied. LOC14 is a potent, non-covalent, and reversible inhibitor for protein disulfide isomerase that has a neuroprotective effect in corticostriatal brain culture, and it was shown that LOC14 was well tolerated at high dose of 20 mg/kg to C57BL/6 j mice in the *in vivo* pharmacokinetic study (82). In addition, LOC14 displayed promising effects against Huntington’s disease (83) and can be used in a new anti-influenza therapeutic strategy (84). Here, we showed that LOC14 inhibits Cho1 activity (Fig. 2 and 3); however, the *in vivo* inhibition is not likely conveyed through Cho1 (Fig. 4). In contrast, CBR-5884 is an inhibitor of phosphoglycerate dehydrogenase, blocking *de novo* serine synthesis in cells, and is selectively toxic to cancer cell lines with high serine biosynthetic activity against melanoma and breast cancer lines (56). Here, CBR-5884 was shown to not only inhibit purified Cho1 (Fig. 2 and 6) but also inhibit live cell growth by acting on the Cho1 *in vivo* (Fig. 3 to 5). To our knowledge, this is the first report showing the antifungal effects of LOC14 and CBR-5884.

CBR-5884 was then determined to be a competitive inhibitor of Cho1 with a *K*_i_ of 1,550 ± 245.6 nM via kinetic analysis (Fig. 6). Interestingly, in non-saturating serine concentrations (Fig. 6C), the kinetic curves decreased in height and shifted to the left in the presence of increasing concentration of CBR-5884, indicating a decreasing *K*_m_ and *V*_max_, mimicking an uncompetitive inhibition where the inhibitor only binds to substrate-bound enzyme complex and, thus, depletes its population. This suggests that CBR-5884 is able to compete with serine by binding CDP-DAG-bound Cho1, and it cannot compete with CDP-DAG to bind to empty Cho1. The *V*_max_ of Cho1 in this study is estimated to be 0.128 ± 0.029 nmol/(μg*min), which is close to 0.088 ± 0.007 nmol/(μg*min) as described in reference (33). Also, it is worth mentioning that the curves where CDP-DAG was held constant and serine was varied follow a sigmoidal shape, which is consistent with previous finding that serine-binding may be cooperative (33), but the underlying mechanism is not clear. One small discrepancy has to be pointed out that the *K*_half_ of serine in this study is determined to be 17.08 ± 4.072 mM, which is four times higher than the 4.17 ± 0.45 mM from reference (33). The increased *K*_half_ may be explained by the presence of DMSO in this study, as DMSO has been shown to increase *K*_m_ in some enzymes (85–87).

CBR-5884 and serine were also docked onto the predicted CDP-DAG-bound Cho1 structure, and CBR-5884 was found to overlap with serine in the pocket (Fig. 6E). A detailed ligand interaction map has shown that CBR-5884 almost shielded the β-phosphorus of the CDP-DAG where the nucleophilic attack occurs (30, 35), and also some CBR-5884 poses directly interact with residues R186 and F190 in Cho1 (Fig. S6), which are part of the putative serine-binding site and are shown to be essential for Cho1 activity (26). This indicates again that CBR-5884 inhibits Cho1 by competing with serine. However, we acknowledge that the molecular docking results presented herein are based on a predicted protein model and are subject to the inherent limitations of *in silico* methods. Future experimental validation will be necessary to fully elucidate the molecular mechanisms behind the inhibition by CBR-5884.

Our data strongly suggest that CBR-5884 inhibits Cho1 *in vivo;* however, Cho1 may not be the only cellular target of CBR-5884. This compound causes cells to become more unmasked than the *cho1*ΔΔ mutant (Fig. 5B). This could be due to sudden drop in PS caused by the compound compared with the *cho1∆∆* mutant, which has adjusted to the change. However, it could also be due to inhibition of another target. In addition, in TLC analysis, it was revealed that CBR-5884 decreased PS and also impacted relative PE levels similar to that seen in a *cho1*ΔΔ mutant (Fig. 5E and F). However, the CBR-5884 compound caused a greater decrease in PE levels in the *cho1∆∆* mutant than that observed in wild-type or untreated *cho1∆∆* cells. The compound also decreases growth of the *cho1*ΔΔ strain in rich YPD media (Fig. S7), suggesting that it has an additional target besides PS synthase. Since the *cho1*ΔΔ strain cannot use PS as the precursor to make PE, the PE is made primarily via the Kennedy pathway, which requires CDP-ethanolamine phosphotransferase (14, 88, 89). Cho1 and CDP-ethanolamine phosphotransferase belong to the same protein family and both use the CDP-alcohol phosphotransferase (CAPT)-binding motif (29, 90, 91), so it is possible that CBR-5884 also inhibits CDP-ethanolamine phosphotransferase activity. Interestingly, PI synthase also has the CAPT motif and binds CDP-DAG (92, 93), but the PI levels were not strongly affected (Fig. 5E and F). Thus, the impact would be more specific to ethanolamine phosphotransferase in this case.

Moreover, this cross-reactivity could potentially be addressed by medicinal chemistry to synthesize analogs more specific for Cho1 and act at lower IC_50_, which could decrease off-target effects and increase potency. Importantly, in this report, we have provided novel proof of principle for successful pharmacological inhibition of a uniquely fungal enzyme central to phospholipid metabolism. Future rational drug design study will optimize CBR-5884 to be more specific for Cho1, as well as increase the solubility and potency of the compound in live cells.

## MATERIALS AND METHODS

### Strain construction and media

This study used a *C. albicans* strain derived from SC5314 that was disrupted for *CHO1* but had the gene complemented back with an affinity-tagged version. This strain *cho1*ΔΔ *P_TEF1_-CHO1-ENLYFQG-HAx3-HISx8* was made in this study and used to solubilize and purify Cho1. This strain expressed a Hisx8-tagged Cho1 protein from a strong, constitutive *P_TEF1_* promoter. To create this strain, a plasmid was generated that carried the tagged *CHO1* gene. This plasmid had a hygromycin B resistance gene, *CaHygB*, as a selectable marker for transformations. To make the plasmid pKE333, first, the *CaHygB* marker cassette was amplified from the *CaHygB*-flipper plasmid (94) using primers YZO113 & YZO114 having *NheI* and *MscI* cut site, respectively (Table 1). The *CaHygB* cassette was ligated into pKE4 plasmid (50) that had been digested with *NheI* and *MscI*, to create the plasmid pKE4-*CaHygB* (pKE333). Then, the *CHO1-ENLYFQG-HAx3-HISx8* gene, with the 3′UTR region, was amplified from pYZ79 (33) using primers YZO110 & YZO111 and ligated into pKE333 cut with *ClaI* and *MluI* to create the plasmid pYZ107. Plasmid pYZ107 was linearized with *PmlI* restriction enzyme (within the *P_TEF1_* sequence) and electroporated into the *cho1*∆∆ strain (14). Transformants were selected on YPD plates containing 600 µg/mL hygromycin B. Colony PCR was performed on six candidates for each gene construct to ensure the successful integration under the *P_TEF1_* promotor on the chromosomal DNA, and no spurious mutations occurred during the transformation. Media used in this study include YPD (1% yeast extract, 2% peptone, 2% dextrose) and minimal medium (0.67% yeast nitrogen base W/O amino acids, 2% dextrose ±1 mM ethanolamine ±50 mM HEPES, pH 7.0, and RPMI +20 mM MOPS pH 7.0).

**TABLE 1:** Primers used in this study

Primer name	Sequence	Purpose
YZO 110	aaaaATCGATATGTCAGACTCATCAGCTACCGGGTTCTCC	F primer to clone CHO1-ENLYFQG-HAx3-HISx8 gene into plasmid pKE333 (ClaI cut site underlined)
YZO 111	aaaaACGCGTACAGAACCAGAATTATTGTTTCAATTGGGA	R primer to clone CHO1-ENLYFQG-HAx3-HISx8 gene into plasmid pKE333 (MluI cut site underlined)
YZO 113	aaaaGCTAGCCGTCAAAACTAGAGAATAATAAAGAAAACG	F primer to clone CaHygB gene into pKE4 to create pKE333 (NheI cut site underlined)
YZO 114	aaaaTGGCCACTGCAGAGGACCACCTTTGATTGTAAATAG	R primer to clone CaHygB gene into pKE4 to create pKE333 (MscI cut site underlined)

### Cell lysis, protein solubilization and purification

The *C. albicans* strain with His-tagged Cho1 expressed from *P_TEF1_* was grown in YPD until the OD_600_ reached 7.0–8.0, and then cells were lysed using a French press, as described in reference (47). Crude membranes were collected and solubilized with 1.5% digitonin as described in reference (33). His-tagged Cho1 was first purified via gravity affinity chromatography as described in reference (33) and was further cleared by size exclusion chromatography [Superdex 200 10/300 Gl column (Cytiva) attached to an NGC chromatography system Quest 10 plus (Bio-Rad)]. The column was equilibrated with 1.5 column volumes of H_2_O and 1.5 column volumes of elution buffer [50 mM Tris-HCl (pH 8.0) +0.04% digitonin] with a flow rate of 0.5 mL/min. Samples loaded onto the above column consisted of a concentrated Cho1 eluted from affinity chromatography that was filtered through 0.22 µm filters that was then manually injected in the sample loop. The injected sample was eluted at a flow rate of 0.4 mL/min. Fractions containing Cho1 were pooled and subjected to AcTEV treatment and then run through another round of affinity chromatography to remove impurities as described in reference (33). The resulting sample was loaded and checked on the blue-native PAGE for purity, oligomer state and homogeneity.

### High-throughput malachite green screen

The bioactives library was purchased from Selleck in 2014, and the remaining libraries (anti-infectives and mechanism of action libraries) were assembled from compounds available at the Chemical Biology and Therapeutics Department at St. Jude Children’s Research Hospital. The anti-infectives library contains a curated list of antimicrobials and antiviral agents (95). The bioactive library has a compilation of compounds acquired from commercial sources and external academic collaborators. The mechanism of action set is a dynamic set of well-annotated compounds with comprehensive coverage of human targets and mechanisms of action (96). All compounds were dissolved in DMSO and 100 nL was transferred to a 384-well clear bottom plate (ThermoFisher Scientific, cat# 265203) using a Beckman Echo 650 acoustic liquid handler. Equal volumes of either the selective CD73 nucleotidase inhibitor AB680 at a final concentration of 1 µM (MedChemExpress, cat# HY-125286) or DMSO were used as positive and negative controls, respectively. A total of 30–35 ng of purified Cho1 protein was used in each reaction in 50 mM Tris-HCl (pH 8.0) for primary screening, combined with 100 µM CDP-DAG (Avanti, cat# 870510), 5 mM serine, 0.4 ng CD73 nucleotidase (R&D systems, cat# EA002), 1 mM MnCl_2_, 0.1% APX-100, and 0.1% digitonin, in a total volume of 10 µL. Counter screen reactions were set up by replacing only the purified Cho1 with 150 µM CMP, with all other components remaining constant. The 10 µL primary and counter screening reactions were delivered to each well of 384-well assay plates containing pre-aliquoted compounds from the library using a Multidrop Combi (ThermoFisher Scientific). This resulted in a DMSO concentration of 1%. After media transfer, assay plates were incubated for 3 h at 30°C. Plates were then removed and 32 µL of malachite green mixture [6 µL malachite A (2%, wt/vol, ammonium molybdate and 20%, vol/vol, sulfuric acid in H_2_O) +20 µL H_2_O + 6 µL malachite B (0.1%, wt/vol, malachite green oxalate and 0.5%, wt/vol, polyvinyl alcohol in H_2_O)] were added to each well via a MultiDrop Combi and incubated for an additional 10 min at room temperature. After incubation, plates were briefly centrifuged at 500 × *g* and absorbance at 620 nm was then measured using a Cytation7 plate reader (Biotek, Winooski, VT). Raw absorbance values of the compounds were normalized to DMSO (0% inhibition) and AB680 (100% inhibition) from both primary and counter screens, and % Δinhibition was calculated by subtracting % inhibition measured in the primary screen from the % inhibition measured in the counter screen. *Z*-factors for each plate were calculated using the in-house program RISE (Robust Investigation of Screening Experiments). All non-PAINs compounds that had >80% Δinhibition progressed to dose-response testing (82 total compounds). Dose-response experiments were performed in triplicate as described above using a 10-point, threefold serial dilution with the top concentration for each compound tested being 200 µM (range: 0.010161–200 μM). The absorbance at 620 nm was then measured using a PHERAstar FS multilabel reader (BMG, Cary, NC). Raw values were once again normalized to DMSO (0% inhibition) and AB680 (100% inhibition) and *Z*-factors for each plate were calculated using RISE. The concentration of test compounds that inhibited Cho1 by 50% (IC_50_ value) as measured by the malachite green assay was computed using nonlinear regression-based fitting of inhibition curves using [inhibitor] vs response-variable slope model in GraphPad Prism version 9.5.0 (GraphPad Software, La Jolla California USA).

### Assay for metabolic labeling of PS using probe C-l-SerN_3_

The C-l-SerN_3_ probe [(*S*)-1-((3-azidopropyl)amino)-3-hydroxy-1-oxopropan-2-aminium chloride] was synthesized as described in reference (59). Cells grown overnight in YPD were washed three times in H_2_O and inoculated into minimal media at a starting OD_600_ of 0.05. Cells were shaken for 5 h before 1.5 mM C-l-SerN_3_ probe was added, along with 170 µM CBR-5884 or an equivalent volume of DMSO. Cells were then incubated for another 5 h before being washed three times with H_2_O, and 5% BSA in 1× PBS was used to treat cells for 20 min. The cells were then washed three times with 1× PBS and then resuspended to OD_600_ of 0.6 in 1 mL PBS + 1 µM AZDye 488 DBCO (Click chemistry tools, cat# 1278-1). Cells were covered with aluminum foil and rocked for 1 h, and the dye was removed by pelleting the cells at 5,000 × *g* followed by washing with shaking at 1,000 rpm three times in 1× PBS, and resuspended in 200 µL Fluoromount-G mounting medium (cat# 00-4958-02). For each treatment, 10 µL of cells was added to a glass slide and 3 µL fresh Fluoromount-G mounting medium was used to mix the sample. Then, a Leica SP8 white light laser confocal microscope was used for imaging. The samples were excited using light at a wavelength of 488 nm, and the resulting fluorescence was captured within the range of 498–550 nm using a HyD detector. The settings for laser strength, gain, and offset were maintained consistently throughout the experiment. Images of treated cells were taken after applying a zoom factor of 3. A total of 40 cells from at least 10 images were used for the quantification in ImageJ software.

### Fluorescence imaging of unmasked β(1-3)-glucan

Wild-type and *cho1*ΔΔ cells were grown in YPD overnight (~16 h), and back diluted to fresh YPD with OD_600_ of ~0.1. The cells were then shaken at 225 rpm for 3 h before 170 µM CBR-5884 or equivalent DMSO were added. The cells were further shaken for 30, 60, 120 min before staining with anti-β(1-3)-glucan antibody. The cells were stained as previously described (57, 97) with the exception that goat anti-mouse antibody conjugated to Alexa Fluor 488 (Jackson ImmunoResearch) was used as the secondary antibody. For imaging, *Candida* cells were resuspended in 200 µL of Fluoromount-G mounting medium and visualized with a Leica SP8 white light laser confocal microscope. The pictures were taken through Leica Application Suite X office software and quantified as described above.

### MIC plates tests and growth curves

Minimum inhibitory concentration (MIC) was determined following Clinical & Lab Standards Institute (CLSI) MIC broth microdilution protocol (Licensed to Todd Reynolds, license # Ord-1138682) (98, 99). Briefly, stock solutions of different compounds (purchased from MedChemExpress) were prepared at a concentration of 10 mM in DMSO and subsequently diluted as required in the same solvent. Wild-type *C. albicans* strain SC5314, as well as four additional wild-type strains—GDH2346, 3153A, TFPY412, and TFPY2307, were cultured overnight in YPD medium at 30°C, washed with water three times, and then diluted to a concentration of 2 × 10^3^ cells/mL (OD_600_ = 0.00008) in one of the three media [minimal medium, minimal medium buffered with 50 mM HEPES (pH 7.0) and RPMI MOPS (pH 7.0)]. The *cho1*ΔΔ strain was also included as a control. A volume of 100 µL from each cell suspension was dispensed into the wells of a flat-bottom 96-well plate. An additional 100 µL of corresponding medium, containing twice the final desired concentration of each drug, was added to each well, leading to a final initial inoculum of 1 × 10^3^ cells/mL (OD_600_ = 0.00004). The final DMSO concentration was maintained at 2.5% across all treatments. Following preparation, the plates were incubated at 37°C for 48 h and photos were taken with a Bio-Rad Gel Doc XR + Imaging System.

Plate assays and growth curves were conducted in minimal media or minimal media supplemented with 1 mM ethanolamine (ETA). Wild-type SC5314 and *cho1*ΔΔ strains were grown in YPD overnight and washed three times with H_2_O before inoculation. The starting OD_600_ is 0.05 for both plate assays and growth curves. For plate assays, both wild-type and *cho1*ΔΔ strains were grown in flat-bottom 96-well plates at a final volume of 200 µL, in the presence of different compounds or DMSO as indicated at 30°C. The final DMSO concentration was maintained at 2.5% across all treatments. A Leica inverted microscope was then used to visualize growth after 24 h incubations. In the meantime, cells grown in different compounds were subjected to live cell counting. Cell cultures from flat-bottom 96-well plates after 24 h incubation were diluted 100–10,000 times before plating on the minimal media, and the total colony-forming unit (CFU) was kept within 200 for each plate. A total of six biological replicates were done in each condition.

The growth curves with LOC14 and CBR-5884 treatments were measured from 2 to 30 h with OD_600_ measurements every 2 h. A final concentration of 170 µM CBR-5884, 430 µM LOC14, and 5 mM serine was added into each group as indicated. To test the stability of CBR-5884, minimal medium supplemented with 170 µM CBR-5884 or equivalent DMSO was agitated for 12 h before the introduction of wildtype *C. albicans* SC5314 strain, and growth curves were recorded from 0 to 14 h with OD_600_ measurement every 2 h. To check the resistance of *C. albicans* in CBR-5884, SC5314 strain was grown in 170 µM CBR-5884 for 12 and 24 h before being diluted in fresh minimal medium supplemented with fresh 170 µM CBR-5884 or equivalent DMSO. The growth curves were recorded from 0 to 14 h with OD_600_ measurement every 2 h. The final DMSO concentration was maintained at 1.7% across all treatments.

### PS synthase assay

Enzymatic activity of Cho1 in the presence of the compounds was measured using the radioactive PS synthase assay. For purified Cho1, the procedure was fully described in reference (33). Briefly, 1–2 μg of purified Cho1 was added to the reaction containing 50 mM Tris-HCl (pH 8.0), 1 mM MnCl_2_, 0.1% Triton X-100, 0.04% digitonin, 0.1 mM (4.7 mol %), 16:0 CDP-DAG (Avanti, cat# 870510), and 0.5 mM L-serine [spiked with 5% (by volume) L-[^3^H]-serine (15 Ci/mmol)] at a total volume of 100 µL, in the presence of 100 µM of each compound or equivalent DMSO solvent. The reaction was conducted at 30°C for 30 min, and [^3^H]PS produced in the reaction was measured using a liquid scintillation counter. For crude membrane samples, crude membrane preps from wildtype and *cho1*ΔΔ strains were collected and assayed as described in reference (33) with the exception that 2 mM L-serine [spiked with 5% (by volume) L-[^3^H]-serine (15 Ci/mmol)] was used in each reaction. A final concentration of 1 mM of each compound was used for the crude membrane samples.

For CBR-5884 washout assays, purified Cho1 was incubated with CBR-5884 at a final concentration of 100 µM, or equivalent DMSO, in 100 µL volume on ice for 2 h. The CBR-5884 was then washed out by exchanging with 4 mL 50 mM Tris-HCl at pH 8.0 and 0.1% digitonin three times in the Amicon Ultra Centrifugal Filter (10 kDa MWCO). The sample was concentrated to the volume of approximately 500 µL, and protein concentration was determined using the Pierce detergent-compatible Bradford assay kit. The reaction was set up with 50 mM Tris-HCl (pH 8.0), 1 mM MnCl_2_, 0.1% Triton X-100, 0.1% digitonin, 0.1 mM (4.7 mol %), 16:0 CDP-DAG (Avanti, cat# 870510), and 10 mM L-serine [spiked with 5% (by volume) L-[3H]-serine (15 Ci/mmol)] at a total volume of 100 µL, in the presence of 100 µM CBR-5884 or equivalent DMSO solvent. The reaction was stopped at 20, 40, and 60 min, and specific activity was calculated based on the slope of linear PS production, representing the initial velocity.

For the kinetic curves, the specific activity was measured in the reaction containing 50 mM Tris-HCl (pH 8.0), 1 mM MnCl_2_, 0.025%–0.3% Triton X-100 and 0.033%–0.07% digitonin with 0.75–1.5 µg purified Cho1 protein. The concentrations of 18:1 CDP-DAG (Avanti, cat# 870520) and serine are indicated in the graphs, and the mol % of CDP-DAG was kept between 4.2% and 5.0% for the curve where CDP-DAG was varied (Fig. 6C and D) and at 4.8% (200 μM) for the curve where serine was varied (Fig. 6B). The concentrations of CBR-5884 used in each reaction are indicated in the graph, and CBR-5884 was incubated with purified Cho1 protein in the presence of CDP-DAG on ice for at least 2 h before the addition of L-[^3^H]-serine. The reaction was stopped at 20, 40, and 60 min, and specific activity was calculated based on the slope of linear PS production, representing the initial velocity.

### Thin-layer chromatography

Wild-type SC5314 and *cho1*ΔΔ strains grown overnight in YPD were inoculated into fresh YPD at OD_600_ of 0.1 and were shaken for another 3 h. Then, 170 µM CBR-5884 or equivalent DMSO solvent was added to both wild-type and *cho1*ΔΔ cultures, and cells were shaken for another 2 h. Then, cells were washed with 1× PBS three times and normalized to a total OD_600_ of 1. The phospholipids were extracted with the hot ethanol method as described in reference (14). A Whatman 250 µm silica gel aluminum backed plate was treated, and separation of phospholipids was carried out as described in reference (100). Phospholipid standards PI, PE, PS, and PC were purchased from Avanti. The quantification of the phospholipids was done in ImageJ software.

### Computational docking

The computational docking was conducted in Molecular Operating Environment software (MOE, Chemical Computing Group, Ltd, Montreal, Canada). A CDP-DAG-bound *C. albicans* Cho1 structure was generated by superposing the *C. albicans* Cho1 AlphaFold model on the CDP-DAG-bound PS synthase from *Methanocaldococcus jannaschii* (PDB: 7B1L) (32). Structures from CBR-5884 and serine were introduced into the structure, and the system was quickly prepped and energy-minimized for docking. Potential docking sites were predicted by “site finder” function in MOE and all the sites having above 0 possibilities are combined for docking (Fig. S5B). Both serine and CBR-5884 molecules were docked into the potential sites 20,000 times, and triangle matcher placement (scored by London dG) and rigid receptor refinement (scored by GBVI/WSA dG) were used to pick the top five poses. The ligand interaction map was also generated in MOE.

### Statistical analysis and molecular weight estimation on the gels

All the statistical analyses were performed with GraphPad Prism 9.1 software. The PS synthase activities were compared using ordinary (equal SDs) or Brown-Forsythe and Welch ANOVA tests (unequal SDs). Blue native PAGE and Coomassie Blue R-250 staining were conducted as described in reference (33), and all MW estimates were conducted in the band analysis tool of the Quantity One software (Bio-Rad).

## Data Availability

The original contributions presented in the study are included in the manuscript/supplementary files; Source data files have been provided for figures and supplemental figures. Further inquiries can be directed to the corresponding author.

## SUPPLEMENTAL MATERIAL

Supplemental Figures mbio.00633-24-s0001.pdf Fig. S1-S7.

ASM does not own the copyrights to Supplemental Material that may be linked to, or accessed through, an article. The authors have granted ASM a non-exclusive, world-wide license to publish the Supplemental Material files. Please contact the corresponding author directly for reuse.

## References

[1] Morrell M, Fraser VJ, Kollef MH. 2005. Delaying the empiric treatment of Candida bloodstream infection until positive blood culture results are obtained: a potential risk factor for hospital mortality. Antimicrob Agents Chemother 49:3640–3645. doi:10.1128/AAC.49.9.3640-3645.2005 16127033 PMC1195428

[2] Wisplinghoff H, Bischoff T, Tallent SM, Seifert H, Wenzel RP, Edmond MB. 2004. Nosocomial bloodstream infections in US hospitals: analysis of 24,179 cases from a prospective nationwide surveillance study. Clin Infect Dis 39:309–317. doi:10.1086/421946 15306996

[3] Patel PK, Erlandsen JE, Kirkpatrick WR, Berg DK, Westbrook SD, Louden C, Cornell JE, Thompson GR, Vallor AC, Wickes BL, Wiederhold NP, Redding SW, Patterson TF. 2012. The changing epidemiology of oropharyngeal candidiasis in patients with HIV/AIDS in the era of antiretroviral therapy. AIDS Res Treat 2012:262471. doi:10.1155/2012/262471 22970352 PMC3434376

[4] Benedict K, Singleton AL, Jackson BR, Molinari NAM. 2022. Survey of incidence, lifetime prevalence, and treatment of self-reported vulvovaginal candidiasis, United States, 2020. BMC Womens Health 22:147. doi:10.1186/s12905-022-01741-x 35538480 PMC9092842

[5] Novosad SA, Fike L, Dudeck MA, Allen-Bridson K, Edwards JR, Edens C, Sinkowitz-Cochran R, Powell K, Kuhar D. 2020. Pathogens causing central-line–associated bloodstream infections in acute-care hospitals—United States, 2011–2017. Infect Control Hosp Epidemiol 41:313–319. doi:10.1017/ice.2019.303 31915083 PMC13108540

[6] Brown GD, Denning DW, Gow NAR, Levitz SM, Netea MG, White TC. 2012. Hidden killers: human fungal infections. Sci Transl Med 4:165rv13. doi:10.1126/scitranslmed.3004404 23253612

[7] Bustamante CI. 2005. Treatment of Candida infection: a view from the trenches! Curr Opin Infect Dis 18:490–495. doi:10.1097/01.qco.0000191516.43792.61 16258321

[8] Kullberg B, Filler S, Calderone R. 2002. Candida and candidiasis. ASM Press, Washington, DC.

[9] Gow NAR, Johnson C, Berman J, Coste AT, Cuomo CA, Perlin DS, Bicanic T, Harrison TS, Wiederhold N, Bromley M, Chiller T, Edgar K. 2022. The importance of antimicrobial resistance in medical mycology. Nat Commun 13:5352. doi:10.1038/s41467-022-32249-5 36097014 PMC9466305

[10] Pfaller M, Neofytos D, Diekema D, Azie N, Meier-Kriesche H-U, Quan S-P, Horn D. 2012. Epidemiology and outcomes of candidemia in 3,648 patients: data from the prospective antifungal therapy (PATH alliance) registry, 2004–2008. Diagn Microbiol Infect Dis 74:323–331. doi:10.1016/j.diagmicrobio.2012.10.003 23102556

[11] Holeman CW, Einstein H. 1963. The toxic effects of amphotericin B in man. Calif Med 99:90–93. 13961286 PMC1515233

[12] Ghannoum MA, Rice LB. 1999. Antifungal agents: mode of action, mechanisms of resistance, and correlation of these mechanisms with bacterial resistance. Clin Microbiol Rev 12:501–517. doi:10.1128/CMR.12.4.501 10515900 PMC88922

[13] Whaley SG, Berkow EL, Rybak JM, Nishimoto AT, Barker KS, Rogers PD. 2016. Azole antifungal resistance in Candida albicans and emerging non-albicans Candida species. Front Microbiol 7:2173. doi:10.3389/fmicb.2016.02173 28127295 PMC5226953

[14] Chen Y, Montedonico AE, Kauffman S, Dunlap JR, Menn F, Reynolds TB. 2010. Phosphatidylserine synthase and phosphatidylserine decarboxylase are essential for cell wall integrity and virulence in Candida albicans . Mol Microbiol 75:1112–1132. doi:10.1111/j.1365-2958.2009.07018.x 20132453

[15] Davis SE, Tams RN, Solis NV, Wagner AS, Chen T, Jackson JW, Hasim S, Montedonico AE, Dinsmore J, Sparer TE, Filler SG, Reynolds TB. 2018. Candida albicans cannot acquire sufficient ethanolamine from the host to support virulence in the absence of de novo phosphatidylethanolamine synthesis. Infect Immun 86:e00815-17. doi:10.1128/IAI.00815-17 29866908 PMC6056879

[16] Konarzewska P, Wang Y, Han G-S, Goh KJ, Gao Y-G, Carman GM, Xue C. 2019. Phosphatidylserine synthesis is essential for viability of the human fungal pathogen Cryptococcus neoformans. J Biol Chem 294:2329–2339. doi:10.1074/jbc.RA118.006738 30602568 PMC6378964

[17] Braun BR, van Het Hoog M, d’Enfert C, Martchenko M, Dungan J, Kuo A, Inglis DO, Uhl MA, Hogues H, Berriman M, et al.. 2005. A human-curated annotation of the Candida albicans genome. PLoS Genet 1:36–57. doi:10.1371/journal.pgen.0010001 16103911 PMC1183520

[18] Kohlwein SD, Kuchler K, Sperka-Gottlieb C, Henry SA, Paltauf F. 1988. Identification of mitochondrial and microsomal phosphatidylserine synthase in Saccharomyces cerevisiae as the gene product of the CHO1 structural gene. J Bacteriol 170:3778–3781. doi:10.1128/jb.170.8.3778-3781.1988 2841305 PMC211363

[19] Kuchler K, Daum G, Paltauf F. 1986. Subcellular and submitochondrial localization of phospholipid-synthesizing enzymes in Saccharomyces cerevisiae. J Bacteriol 165:901–910. doi:10.1128/jb.165.3.901-910.1986 3005242 PMC214514

[20] Gaigg B, Simbeni R, Hrastnik C, Paltauf F, Daum G. 1995. Characterization of a microsomal subfraction associated with mitochondria of the yeast, Saccharomyces cerevisiae. Involvement in synthesis and import of phospholipids into mitochondria. Biochim Biophys Acta 1234:214–220. doi:10.1016/0005-2736(94)00287-y 7696296

[21] Poole MA, Homann MJ, Bae-Lee MS, Carman GM. 1986. Regulation of phosphatidylserine synthase from Saccharomyces cerevisiae by phospholipid precursors. J Bacteriol 168:668–672. doi:10.1128/jb.168.2.668-672.1986 3023284 PMC213533

[22] Carson MA, Atkinson KD, Waechter CJ. 1982. Properties of particulate and solubilized phosphatidylserine synthase activity from Saccharomyces cerevisiae. inhibitory effect of choline in the growth medium. J Biol Chem 257:8115–8121. doi:10.1016/S0021-9258(18)34304-7 6282872

[23] Letts VA, Henry SA. 1985. Regulation of phospholipid synthesis in phosphatidylserine synthase-deficient (chol) mutants of Saccharomyces cerevisiae. J Bacteriol 163:560–567. doi:10.1128/jb.163.2.560-567.1985 2991194 PMC219158

[24] Letts VA, Klig LS, Bae-Lee M, Carman GM, Henry SA. 1983. Isolation of the yeast structural gene for the membrane-associated enzyme phosphatidylserine synthase. Proc Natl Acad Sci U S A 80:7279–7283. doi:10.1073/pnas.80.23.7279 6316353 PMC390038

[25] Bae-Lee MS, Carman GM. 1984. Phosphatidylserine synthesis in Saccharomyces cerevisiae. purification and characterization of membrane-associated phosphatidylserine synthase. J Biol Chem 259:10857–10862. doi:10.1016/S0021-9258(18)90592-2 6088519

[26] Zhou Y, Cassilly CD, Reynolds TB. 2021. Mapping the substrate-binding sites in the phosphatidylserine synthase in Candida albicans. Front Cell Infect Microbiol 11:765266. doi:10.3389/fcimb.2021.765266 35004345 PMC8727905

[27] Nikawa J, Tsukagoshi Y, Kodaki T, Yamashita S. 1987. Nucleotide sequence and characterization of the yeast PSS gene encoding phosphatidylserine synthase. Eur J Biochem 167:7–12. doi:10.1111/j.1432-1033.1987.tb13297.x 3040403

[28] Hjelmstad RH, Bell RM. 1991. sn-1,2-diacylglycerol choline- and ethanolaminephosphotransferases in Saccharomyces cerevisiae. Nucleotide sequence of the EPT1 gene and comparison of the CPT1 and EPT1 gene products. J Biol Chem 266:5094–5103. doi:10.1016/S0021-9258(19)67760-4 1848238

[29] Hjelmstad RH, Bell RM. 1990. The sn-1,2-diacylglycerol cholinephosphotransferase of Saccharomyces cerevisiae. Nucleotide sequence, transcriptional mapping, and gene product analysis of the CPT1 gene. J Biol Chem 265:1755–1764. doi:10.1016/S0021-9258(19)40081-1 2153142

[30] Grāve K, Bennett MD, Högbom M. 2019. Structure of Mycobacterium tuberculosis phosphatidylinositol phosphate synthase reveals mechanism of substrate binding and metal catalysis. Commun Biol 2:175. doi:10.1038/s42003-019-0427-1 31098408 PMC6506517

[31] Zhou Y, Reynolds T. 2021. Identification of the substrate-binding sites in the phosphatidylserine synthase from Candida albicans. FASEB J 35. doi:10.1096/fasebj.2021.35.S1.02666 PMC8727905 35004345

[32] Centola M, van Pee K, Betz H, Yildiz Ö. 2021. Crystal structures of phosphatidyl serine synthase PSS reveal the catalytic mechanism of CDP-DAG alcohol O-phosphatidyl transferases. Nat Commun 12:6982. doi:10.1038/s41467-021-27281-w 34848707 PMC8633023

[33] Zhou Y, Syed JH, Semchonok DA, Wright E, Kyrilis FL, Hamdi F, Kastritis PL, Bruce BD, Reynolds TB. 2023. Solubilization, purification, and characterization of the hexameric form of phosphatidylserine synthase from. J Biol Chem 299:104756. doi:10.1016/j.jbc.2023.104756 37116705 PMC10248529

[34] Nogly P, Gushchin I, Remeeva A, Esteves AM, Borges N, Ma P, Ishchenko A, Grudinin S, Round E, Moraes I, Borshchevskiy V, Santos H, Gordeliy V, Archer M. 2014. X-ray structure of a CDP-alcohol phosphatidyltransferase membrane enzyme and insights into its catalytic mechanism. Nat Commun 5:4169. doi:10.1038/ncomms5169 24942835

[35] Sciara G, Clarke OB, Tomasek D, Kloss B, Tabuso S, Byfield R, Cohn R, Banerjee S, Rajashankar KR, Slavkovic V, Graziano JH, Shapiro L, Mancia F. 2014. Structural basis for catalysis in a CDP-alcohol phosphotransferase. Nat Commun 5:4068. doi:10.1038/ncomms5068 24923293 PMC4098843

[36] Clarke OB, Tomasek D, Jorge CD, Dufrisne MB, Kim M, Banerjee S, Rajashankar KR, Shapiro L, Hendrickson WA, Santos H, Mancia F. 2015. Structural basis for phosphatidylinositol-phosphate biosynthesis. Nat Commun 6:8505. doi:10.1038/ncomms9505 26510127 PMC4634129

[37] Belcher Dufrisne M, Jorge CD, Timóteo CG, Petrou VI, Ashraf KU, Banerjee S, Clarke OB, Santos H, Mancia F. 2020. Structural and functional characterization of phosphatidylinositol-phosphate biosynthesis in mycobacteria. J Mol Biol 432:5137–5151. doi:10.1016/j.jmb.2020.04.028 32389689 PMC7483940

[38] Wang L, Zhou M. 2023. Structure of a eukaryotic cholinephosphotransferase-1 reveals mechanisms of substrate recognition and catalysis. Nat Commun 14:2753. doi:10.1038/s41467-023-38003-9 37179328 PMC10182977

[39] Wang Z, Yang M, Yang Y, He Y, Qian H. 2023. Structural basis for catalysis of human choline/ethanolamine phosphotransferase 1. Nat Commun 14:2529. doi:10.1038/s41467-023-38290-2 37137909 PMC10156783

[40] Yang B, Yao H, Li D, Liu Z. 2021. The phosphatidylglycerol phosphate synthase PgsA utilizes a trifurcated amphipathic cavity for catalysis at the membrane-cytosol interface. Curr Res Struct Biol 3:312–323. doi:10.1016/j.crstbi.2021.11.005 34901881 PMC8640168

[41] Nikawa J-I, Yamashita S. 1981. Characterization of phosphatidylserine synthase from Saccharomyces cerevisiae and a mutant defective in the enzyme. Biochim Biophys Acta 665:420–426. doi:10.1016/0005-2760(81)90254-x 6271228

[42] Carman GM, Matas J. 1981. Solubilization of microsomal-associated phosphatidylserine synthase and phosphatidylinositol synthase from Saccharomyces cerevisiae. Can J Microbiol 27:1140–1149. doi:10.1139/m81-179 6274497

[43] Kiyono K, Miura K, Kushima Y, Hikiji T, Fukushims M, Shibuya I, Ohta A. 1987. Primary structure and product characterization of the Saccharomyces cerevisiae CHO1 gene that encodes phosphatidylserine synthase. J Biochem 102:1089–1100. doi:10.1093/oxfordjournals.jbchem.a122147 2830250

[44] Mandal S, Moudgil M, Mandal SK. 2009. Rational drug design. Eur J Pharmacol 625:90–100. doi:10.1016/j.ejphar.2009.06.065 19835861

[45] Cassilly CD, Maddox MM, Cherian PT, Bowling JJ, Hamann MT, Lee RE, Reynolds TB. 2016. SB-224289 antagonizes the antifungal mechanism of the marine depsipeptide papuamide A. PLoS One 11:e0154932. doi:10.1371/journal.pone.0154932 27183222 PMC4868317

[46] Pokharel M, Konarzewska P, Roberge JY, Han G-S, Wang Y, Carman GM, Xue C. 2022. The anticancer drug bleomycin shows potent antifungal activity by altering phospholipid biosynthesis. Microbiol Spectr 10:e0086222. doi:10.1128/spectrum.00862-22 36036637 PMC9602507

[47] Cassilly CD, Farmer AT, Montedonico AE, Smith TK, Campagna SR, Reynolds TB. 2017. Role of phosphatidylserine synthase in shaping the phospholipidome of Candida albicans. FEMS Yeast Res 17:fox007. doi:10.1093/femsyr/fox007 28158422 PMC5399917

[48] Zhou Y, Bruce B, Reynolds T. 2022. Solubilization and purification of phosphatidylserine synthase from Candida albicans. FASEB J 36. doi:10.1096/fasebj.2022.36.S1.L7431 PMC10248529 37116705

[49] Wu ZL, Ethen CM, Prather B, Machacek M, Jiang W. 2011. Universal phosphatase-coupled glycosyltransferase assay. Glycobiology 21:727–733. doi:10.1093/glycob/cwq187 21081508

[50] Willems HME, Bruner WS, Barker KS, Liu J, Palmer GE, Peters BM. 2017. Overexpression of Candida albicans secreted aspartyl proteinase 2 or 5 is not sufficient for exacerbation of immunopathology in a murine model of vaginitis. Infect Immun 85:e00248-17. doi:10.1128/IAI.00248-17 28760935 PMC5607425

[51] Lawson KV, Kalisiak J, Lindsey EA, Newcomb ET, Leleti MR, Debien L, Rosen BR, Miles DH, Sharif EU, Jeffrey JL, et al.. 2020. Discovery of AB680: a potent and selective inhibitor of CD73. J Med Chem 63:11448–11468. doi:10.1021/acs.jmedchem.0c00525 32614585

[52] Zhang J-H, Chung TDY, Oldenburg KR. 1999. A simple statistical parameter for use in evaluation and validation of high throughput screening assays. J Biomol Screen 4:67–73. doi:10.1177/108705719900400206 10838414

[53] Baell JB, Nissink JWM. 2018. Seven year itch: pan-assay interference compounds (PAINS) in 2017—utility and limitations. ACS Chem Biol 13:36–44. doi:10.1021/acschembio.7b00903 29202222 PMC5778390

[54] Willcox MD, Webb BC, Thakur A, Harty DW. 1998. Interactions between Candida species and platelets. J Med Microbiol 47:103–110. doi:10.1099/00222615-47-2-103 9879951

[55] Vargas K, Wertz PW, Drake D, Morrow B, Soll DR. 1994. Differences in adhesion of Candida albicans 3153A cells exhibiting switch phenotypes to buccal epithelium and stratum corneum. Infect Immun 62:1328–1335. doi:10.1128/iai.62.4.1328-1335.1994 8132340 PMC186281

[56] Mullarky E, Lucki NC, Beheshti Zavareh R, Anglin JL, Gomes AP, Nicolay BN, Wong JCY, Christen S, Takahashi H, Singh PK, Blenis J, Warren JD, Fendt S-M, Asara JM, DeNicola GM, Lyssiotis CA, Lairson LL, Cantley LC. 2016. Identification of a small molecule inhibitor of 3-phosphoglycerate dehydrogenase to target serine biosynthesis in cancers. Proc Natl Acad Sci U S A 113:1778–1783. doi:10.1073/pnas.1521548113 26831078 PMC4763784

[57] Davis SE, Hopke A, Minkin SC, Montedonico AE, Wheeler RT, Reynolds TB. 2014. Masking of β(1-3)-glucan in the cell wall of Candida albicans from detection by innate immune cells depends on phosphatidylserine. Infect Immun 82:4405–4413. doi:10.1128/IAI.01612-14 25114110 PMC4187869

[58] Weber RWS. 2002. Vacuoles and the fungal lifestyle. Mycologist 16:10–20. doi:10.1017/S0269915X02006110

[59] Ancajas CF, Alam S, Alves DS, Zhou Y, Wadsworth NM, Cassilly CD, Ricks TJ, Carr AJ, Reynolds TB, Barrera FN, Best MD. 2023. Cellular labeling of phosphatidylserine using clickable serine probes. ACS Chem Biol 18:377–384. doi:10.1021/acschembio.2c00813 36745020 PMC13332399

[60] Ancajas CF, Carr AJ, Lou J, Sagar R, Zhou Y, Reynolds TB, Best MD. 2023. Harnessing clickable acylated glycerol probes as chemical tools for tracking glycerolipid metabolism. Chemistry 29:e202300417. doi:10.1002/chem.202300417 37085958 PMC10498425

[61] Antonsson BE. 1994. Purification and characterization of phosphatidylinositol synthase from human placenta. Biochem J 297 (Pt 3):517–522. doi:10.1042/bj2970517 8110188 PMC1137864

[62] Antonioli L, Pacher P, Vizi ES, Haskó G. 2013. CD39 and CD73 in immunity and inflammation. Trends Mol Med 19:355–367. doi:10.1016/j.molmed.2013.03.005 23601906 PMC3674206

[63] Cardoso AM, Schetinger MRC, Correia-de-Sá P, Sévigny J. 2015. Impact of ectonucleotidases in autonomic nervous functions. Auton Neurosci 191:25–38. doi:10.1016/j.autneu.2015.04.014 26008223

[64] Duarte-Araújo M, Nascimento C, Timóteo MA, Magalhães-Cardoso MT, Correia-de-Sá P. 2009. Relative contribution of ecto-ATPase and ecto-ATPDase pathways to the biphasic effect of ATP on acetylcholine release from myenteric motoneurons. Br J Pharmacol 156:519–533. doi:10.1111/j.1476-5381.2008.00058.x 19154428 PMC2697673

[65] Maiorino M, Roveri A, Ursini F. 1992. Antioxidant effect of ebselen (PZ 51): peroxidase mimetic activity on phospholipid and cholesterol hydroperoxides vs free radical scavenger activity. Arch Biochem Biophys 295:404–409. doi:10.1016/0003-9861(92)90534-4 1586168

[66] Maiorino M, Roveri A, Coassin M, Ursini F. 1988. Kinetic mechanism and substrate specificity of glutathione peroxidase activity of ebselen (PZ51). Biochem Pharmacol 37:2267–2271. doi:10.1016/0006-2952(88)90591-6 3377822

[67] Parnham M, Sies H. 2000. Ebselen: prospective therapy for cerebral ischaemia. Expert Opin Investig Drugs 9:607–619. doi:10.1517/13543784.9.3.607 11060699

[68] Liang Q, Shen N, Lai B, Xu C, Sun Z, Wang Z, Li S. 2019. Electrical stimulation degenerated cochlear synapses through oxidative stress in neonatal cochlear explants. Front Neurosci 13:1073. doi:10.3389/fnins.2019.01073 31680814 PMC6803620

[69] Haritha CV, Sharun K, Jose B. 2020. Ebselen, a new candidate therapeutic against SARS-CoV-2. Int J Surg 84:53–56. doi:10.1016/j.ijsu.2020.10.018 33120196 PMC7583587

[70] Nakamura Y, Feng Q, Kumagai T, Torikai K, Ohigashi H, Osawa T, Noguchi N, Niki E, Uchida K. 2002. Ebselen, a glutathione peroxidase mimetic seleno-organic compound, as a multifunctional antioxidant. implication for inflammation-associated carcinogenesis. J Biol Chem 277:2687–2694. doi:10.1074/jbc.M109641200 11714717

[71] Jin Z, Du X, Xu Y, Deng Y, Liu M, Zhao Y, Zhang B, Li X, Zhang L, Peng C, et al.. 2020. Structure of M^pro^ from SARS-CoV-2 and discovery of its inhibitors. Nature 582:289–293. doi:10.1038/s41586-020-2223-y 32272481

[72] Venturini TP, Chassot F, Loreto ÉS, Keller JT, Azevedo MI, Zeni G, Santurio JM, Alves SH. 2016. Antifungal activities of diphenyl diselenide and ebselen alone and in combination with antifungal agents against Fusarium spp. Med Mycol 54:550–555. doi:10.1093/mmy/myv120 26773133

[73] Thangamani S, Eldesouky HE, Mohammad H, Pascuzzi PE, Avramova L, Hazbun TR, Seleem MN. 2017. Ebselen exerts antifungal activity by regulating glutathione (GSH) and reactive oxygen species (ROS) production in fungal cells. Biochim Biophys Acta Gen Subj 1861:3002–3010. doi:10.1016/j.bbagen.2016.09.029 27712973 PMC5148707

[74] Sakita KM, Capoci IRG, Conrado PCV, Rodrigues-Vendramini FAV, Faria DR, Arita GS, Becker TCA, Bonfim-Mendonça P de S, Svidzinski TIE, Kioshima ES. 2021. Efficacy of ebselen against invasive aspergillosis in a murine model. Front Cell Infect Microbiol 11:684525. doi:10.3389/fcimb.2021.684525 34249777 PMC8260993

[75] Marshall AC, Kidd SE, Lamont-Friedrich SJ, Arentz G, Hoffmann P, Coad BR, Bruning JB. 2019. Structure, mechanism, and inhibition of Aspergillus fumigatus thioredoxin reductase. Antimicrob Agents Chemother 63:e02281-18. doi:10.1128/AAC.02281-18 30642940 PMC6395915

[76] Marshall MO, Kates M. 1974. Biosynthesis of nitrogenous phospholipids in spinach leaves. Can J Biochem 52:469–482. doi:10.1139/o74-071 4367462

[77] Billack B, Pietka-Ottlik M, Santoro M, Nicholson S, Młochowski J, Lau-Cam C. 2010. Evaluation of the antifungal and plasma membrane H^+^-ATPase inhibitory action of ebselen and two ebselen analogs in S. cerevisiae cultures. J Enzyme Inhib Med Chem 25:312–317. doi:10.3109/14756360903179419 20210698

[78] Chan G, Hardej D, Santoro M, Lau-Cam C, Billack B. 2007. Evaluation of the antimicrobial activity of ebselen: role of the yeast plasma membrane H^+^-ATPase. J Biochem Mol Toxicol 21:252–264. doi:10.1002/jbt.20189 17912695

[79] Azad GK, Singh V, Mandal P, Singh P, Golla U, Baranwal S, Chauhan S, Tomar RS. 2014. Ebselen induces reactive oxygen species (ROS)-mediated cytotoxicity in Saccharomyces cerevisiae with inhibition of glutamate dehydrogenase being a target. FEBS Open Bio 4:77–89. doi:10.1016/j.fob.2014.01.002 PMC3907691 24490132

[80] Azad GK, Balkrishna SJ, Sathish N, Kumar S, Tomar RS. 2012. Multifunctional ebselen drug functions through the activation of DNA damage response and alterations in nuclear proteins. Biochem Pharmacol 83:296–303. doi:10.1016/j.bcp.2011.10.011 22027221

[81] Terentis AC, Freewan M, Sempértegui Plaza TS, Raftery MJ, Stocker R, Thomas SR. 2010. The selenazal drug ebselen potently inhibits indoleamine 2,3-dioxygenase by targeting enzyme cysteine residues. Biochemistry 49:591–600. doi:10.1021/bi901546e 20000778

[82] Kaplan A, Gaschler MM, Dunn DE, Colligan R, Brown LM, Palmer AG III, Lo DC, Stockwell BR. 2015. Small molecule-induced oxidation of protein disulfide isomerase is neuroprotective. Proc Natl Acad Sci U S A 112:E2245–E2252. doi:10.1073/pnas.1500439112 25848045 PMC4418888

[83] Zhou X, Li G, Kaplan A, Gaschler MM, Zhang X, Hou Z, Jiang M, Zott R, Cremers S, Stockwell BR, Duan W. 2018. Small molecule modulator of protein disulfide isomerase attenuates mutant huntingtin toxicity and inhibits endoplasmic reticulum stress in a mouse model of Huntington’s disease. Human Mol Gen 27:1545–1555. doi:10.1093/hmg/ddy061 PMC5905666 29462355

[84] Chamberlain N, Korwin-Mihavics BR, Nakada EM, Bruno SR, Heppner DE, Chapman DG, Hoffman SM, van der Vliet A, Suratt BT, Dienz O, Alcorn JF, Anathy V. 2019. Lung epithelial protein disulfide isomerase A3 (PDIA3) plays an important role in influenza infection, inflammation, and airway mechanics. Redox Biol 22:101129. doi:10.1016/j.redox.2019.101129 30735910 PMC6365984

[85] Chen QX, Liu XD, Huang H. 2003. Inactivation kinetics of mushroom tyrosinase in the dimethyl sulfoxide solution. Biochemistry (Mosc) 68:644–649. doi:10.1023/A:1024665709631 12943509

[86] Milčić N, Stepanić V, Crnolatac I, Findrik Blažević Z, Brkljača Z, Majerić Elenkov M. 2022. Inhibitory effect of DMSO on halohydrin dehalogenase: experimental and computational insights into the influence of an organic co-solvent on the structural and catalytic properties of a biocatalyst. Chemistry 28:e202201923. doi:10.1002/chem.202201923 35997008

[87] Ostermeier L, Oliva R, Winter R. 2020. The multifaceted effects of DMSO and high hydrostatic pressure on the kinetic constants of hydrolysis reactions catalyzed by α-chymotrypsin. Phys Chem Chem Phys 22:16325–16333. doi:10.1039/d0cp03062g 32648563

[88] Cassilly CD, Reynolds TB. 2018. PS, it’s complicated: the roles of phosphatidylserine and phosphatidylethanolamine in the pathogenesis of Candida albicans and other microbial pathogens. J Fungi (Basel) 4:28. doi:10.3390/jof4010028 29461490 PMC5872331

[89] Zhou Y, Reynolds TB. 2024. Innovations in antifungal drug discovery among cell envelope synthesis enzymes through structural insights. J Fungi 10:171. doi:10.3390/jof10030171 PMC10970773 38535180

[90] McMaster CR, Bell RM. 1994. Phosphatidylcholine biosynthesis in Saccharomyces cerevisiae. regulatory insights from studies employing null and chimeric sn-1,2-diacylglycerol choline- and ethanolaminephosphotransferases. J Biol Chem 269:28010–28016. doi:10.1016/S0021-9258(18)46888-3 7961735

[91] Williams JG, McMaster CR. 1998. Scanning alanine mutagenesis of the CDP-alcohol phosphotransferase motif of Saccharomyces cerevisiae cholinephosphotransferase. J Biol Chem 273:13482–13487. doi:10.1074/jbc.273.22.13482 9593682

[92] Paulus H, Kennedy EP. 1960. The enzymatic synthesis of inositol monophosphatide. J Biol Chem 235:1303–1311. doi:10.1016/S0021-9258(18)69403-7 14431034

[93] Fischl AS, Carman GM. 1983. Phosphatidylinositol biosynthesis in Saccharomyces cerevisiae: purification and properties of microsome-associated phosphatidylinositol synthase. J Bacteriol 154:304–311. doi:10.1128/jb.154.1.304-311.1983 6300035 PMC217460

[94] Liu J, Vogel AK, Miao J, Carnahan JA, Lowes DJ, Rybak JM, Peters BM. 2022. Rapid hypothesis testing in Candida albicans clinical isolates using a cloning-free, modular, and recyclable system for CRISPR-Cas9 mediated mutant and revertant construction. Microbiol Spectr 10:e0263021. doi:10.1128/spectrum.02630-21 35612314 PMC9241802

[95] Nishiguchi G, Das S, Ochoada J, Long H, Lee RE, Rankovic Z, Shelat AA. 2021. Evaluating and evolving a screening library in academia: the St Jude approach. Drug Discov Today 26:1060–1069. doi:10.1016/j.drudis.2021.01.005 33453364 PMC8131249

[96] Canham SM, Wang Y, Cornett A, Auld DS, Baeschlin DK, Patoor M, Skaanderup PR, Honda A, Llamas L, Wendel G, et al.. 2020. Systematic chemogenetic library assembly. Cell Chem Biol 27:1124–1129. doi:10.1016/j.chembiol.2020.07.004 32707038

[97] Chen T, Jackson JW, Tams RN, Davis SE, Sparer TE, Reynolds TB. 2019. Exposure of Candida albicans β (1,3)-glucan is promoted by activation of the Cek1 pathway. PLoS Genet. 15:e1007892. doi:10.1371/journal.pgen.1007892 30703081 PMC6372213

[98] Clinical and Laboratory Standards Institute. 2008. Reference method for broth dilution antifungal susceptibility testing of yeasts. Clinical and Laboratory Standards Institute, Wayne, PA.

[99] Wayne P. 2008. Reference method for broth dilution antifungal susceptibility testing of yeasts. Approved Standard 3:6–12.

[100] Vaden DL, Gohil VM, Gu Z, Greenberg ML. 2005. Separation of yeast phospholipids using one-dimensional thin-layer chromatography. Anal Biochem 338:162–164. doi:10.1016/j.ab.2004.11.020 15707948

