# Supplementary material for: The small molecule CBR-5884 inhibits the Candida albicans phosphatidylserine synthase
Source: mBio. 2024 Apr 9;15(5):e00633-24. doi: 10.1128/mbio.00633-24 (PMC11077991; doi:10.1128/mbio.00633-24)
Supplement: Supplemental Figures — Fig. S1-S7. [file mbio.00633-24-s0001.pdf]

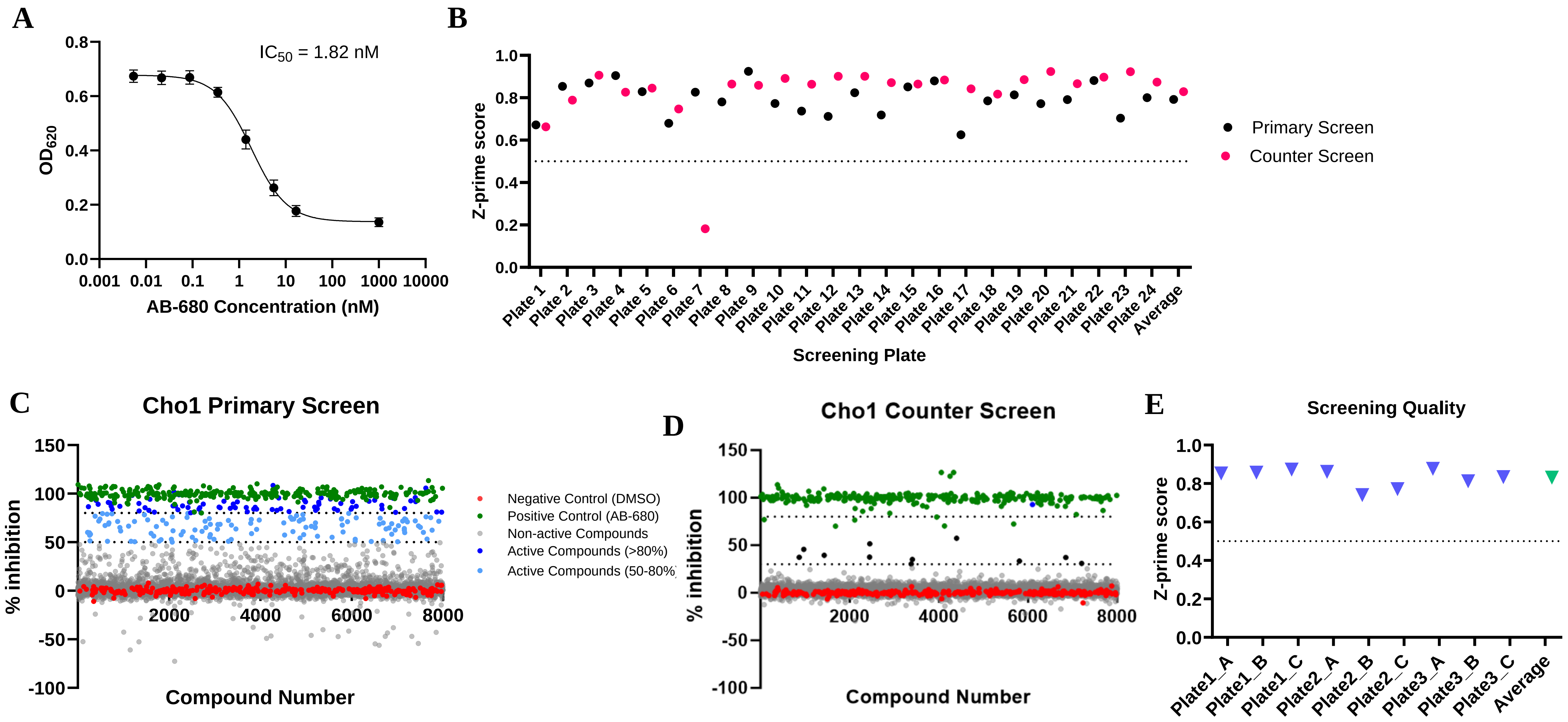

Figure S1. (A) Dose-response curve of AB-680 compound. The dots represent the mean of nine biological replicates, and the error bars are ± standard deviation (S.D.) values. (B) Z-prime scores of plates in the primary and counter screens. (C,D) The dot plots of % inhibition for all the compounds, including controls, used in the primary screen (C) and counter screen (D). Reactions with DMSO and AB-680 were used as 0% inhibition (negative) and 100% inhibition (positive) controls, respectively. The two dotted lines from the Y-axis indicate 80 and 50% inhibition, respectively. (E) Z-prime scores of plates in the dose-response curves for the second-round screen.

Figure S2. (A) Avasimibe, tideglusib and CBR-5884 precipitated at high concentrations; (B, C) The growth rate (h^{-1}) (B) and lag time (h) (C) were calculated from Figure 4D using the logistic growth model from GraphPad Prism. Statistics were conducted using one-way ANOVA and Tukey's multiple comparisons test (ns=not significant, $p > 0.05$; *, $0.05 > p > 0.01$, ****, $0.0001 > p$).

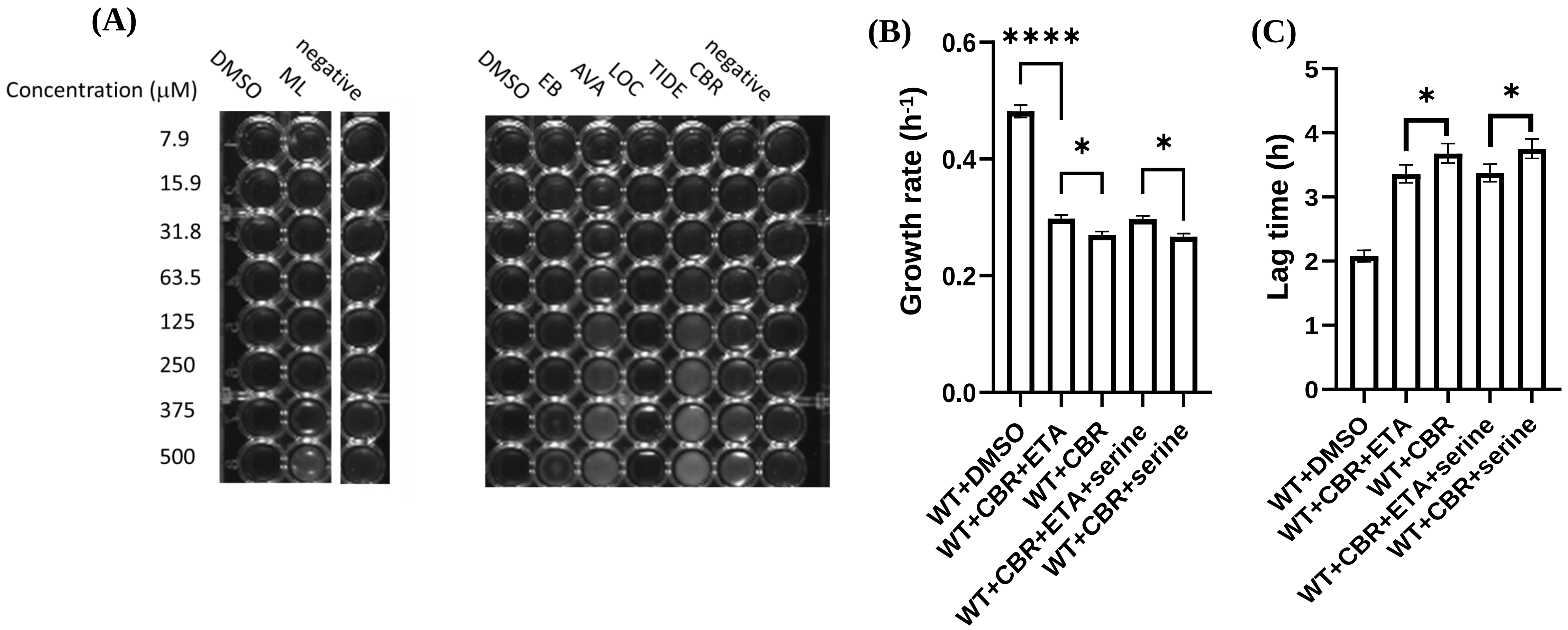

Figure S3. The PS synthase activity of crude membrane containing Cho1 was measured by L-[^{3}H]-serine incorporation into PS in the presence of different inhibitors at 1 mM, and are presented as nmol/(μg protein*min). Statistics were conducted using one-way ANOVA and Dunnett's T3 multiple comparisons test (**, 0.01> p > 0.001; ***, 0.001> p > 0.0001; ****, 0.0001> p). The activities were measured in duplicate with a total of six biological replicates as indicated. The bars represent the mean values and the error bars are ± S.D. values.

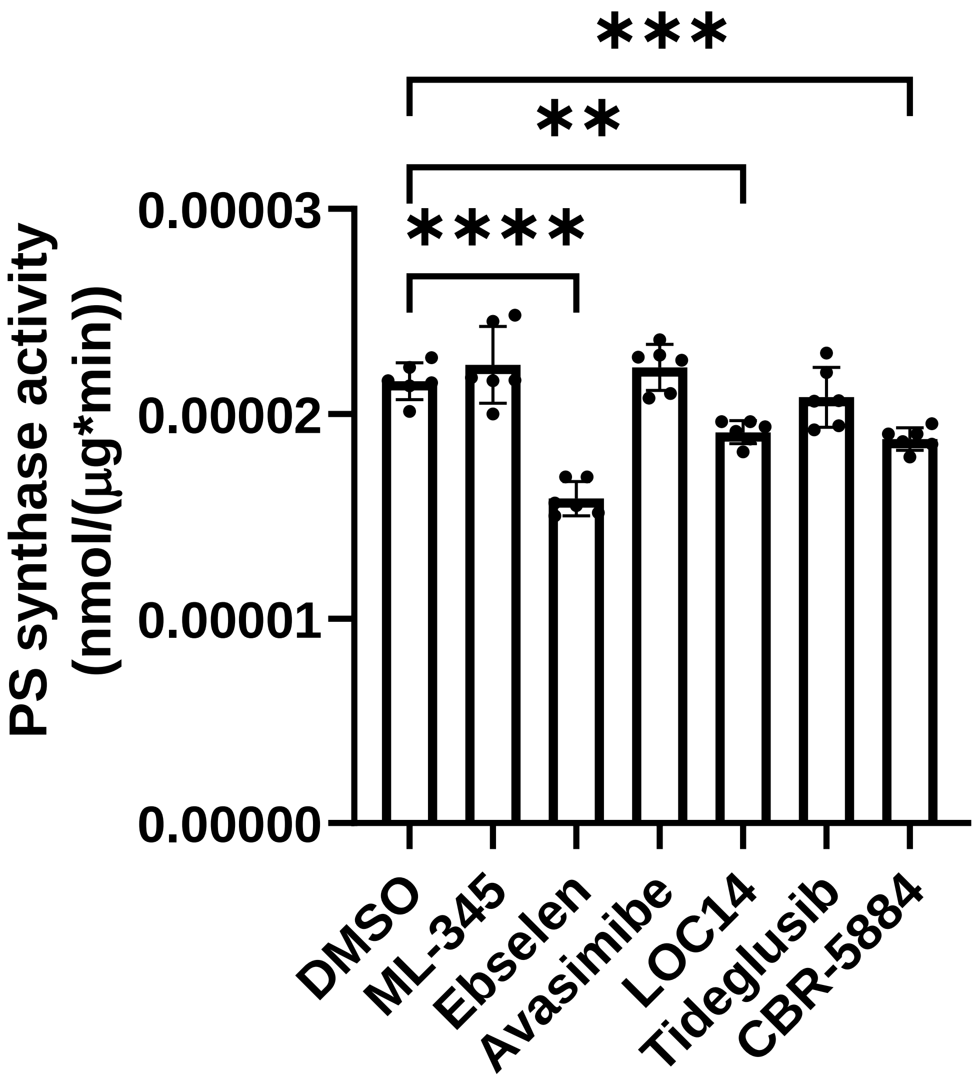

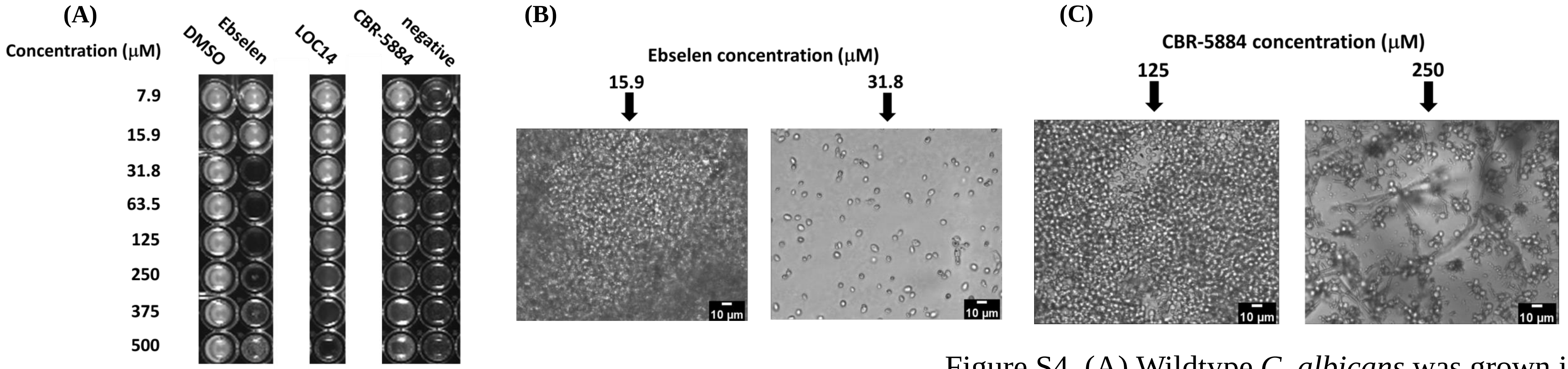

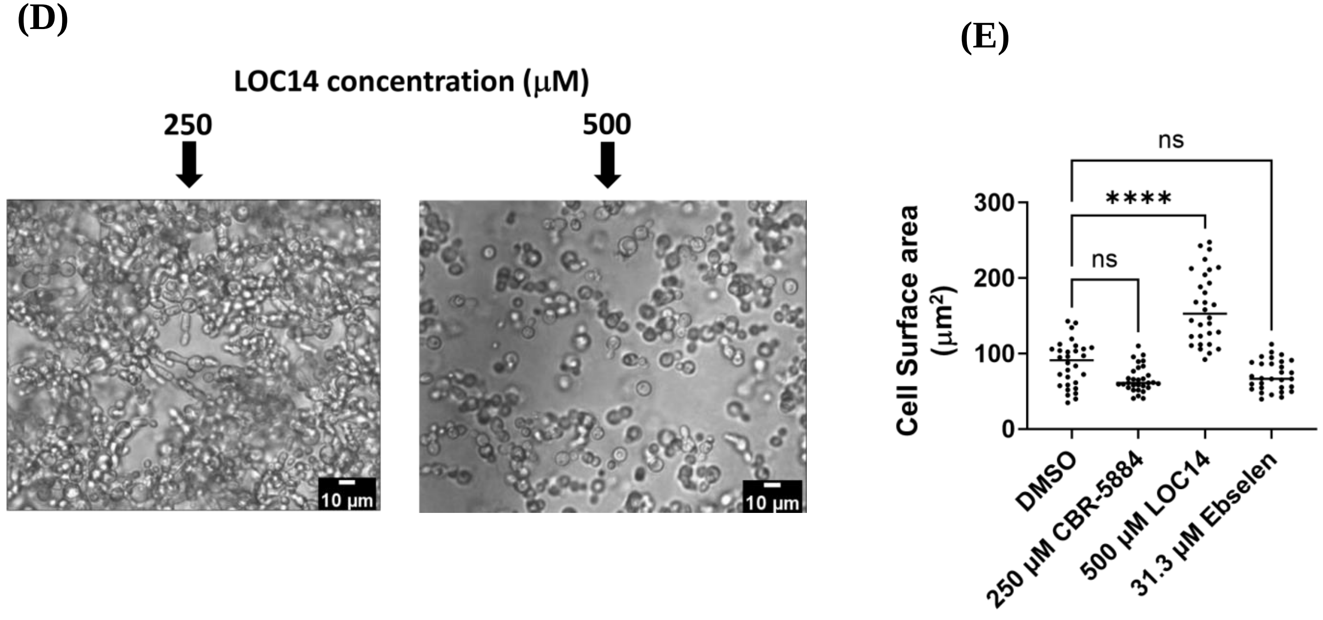

Figure S4. (A) Wildtype *C. albicans* was grown in the minimal medium for 24 hours with DMSO, ebselen, LOC14, and CBR-5884 at the concentrations indicated, and the *cho1*ΔΔ strain in DMSO was used as a control (shown as "negative"). (B-D) Inverted microscopy (630x) was used to visualize cells treated with (B) ebselen (C) CBR-5884 and (D) LOC14 and under the indicated concentrations for 24 hours. Black arrows indicate from which concentrations of compounds the images are taken. (E) The surface area (μm^2) of the cell was quantified for forty cells from three different photographs for each specified treatment using the ImageJ software. Statistics were conducted using one-way ANOVA using Tukey's multiple comparisons test (ns=not significant, ****, $p < 0.0001$)

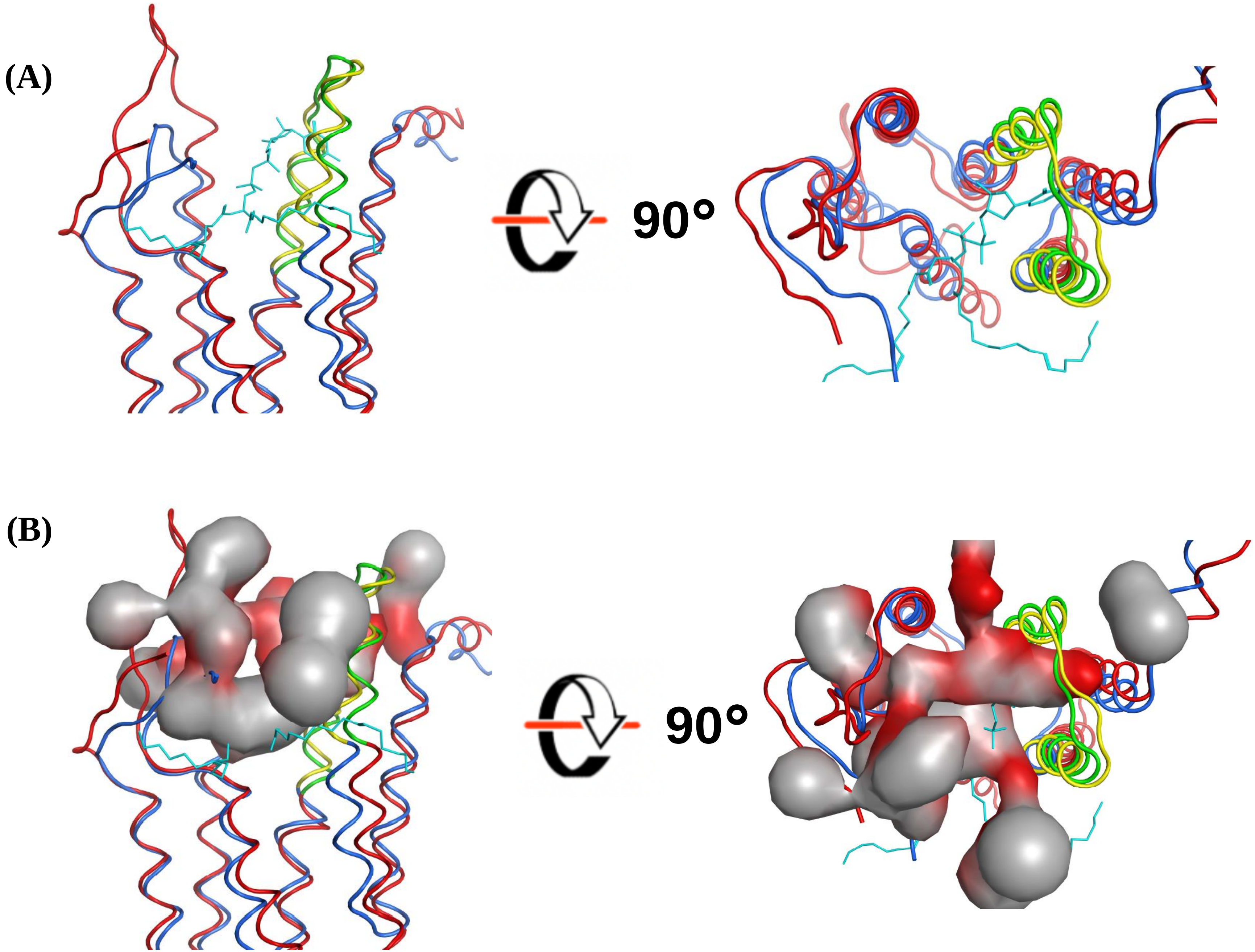

Figure S5. (A) Superposition of *C. albicans* AlphaFold Cho1 model (red) with *Methanocaldococcus jannaschii* PS synthase (blue) (PDB: 7B1L).The conserved CAPT site are highlighted as green and yellow, respectively. CDP-DAG (cyan) is *Methanocaldococcus jannaschii* PS synthase (PDB: 7B1L). (B) All the possible active site pockets predicted from *C. albicans* Cho1 using MOE are shown as pocket surfaces.

CBR-5884 MOE(S) = -11.48 ± 0.12 kcal/mol
Serine MOE(S) = -6.72 ± 0.08 kcal/mol

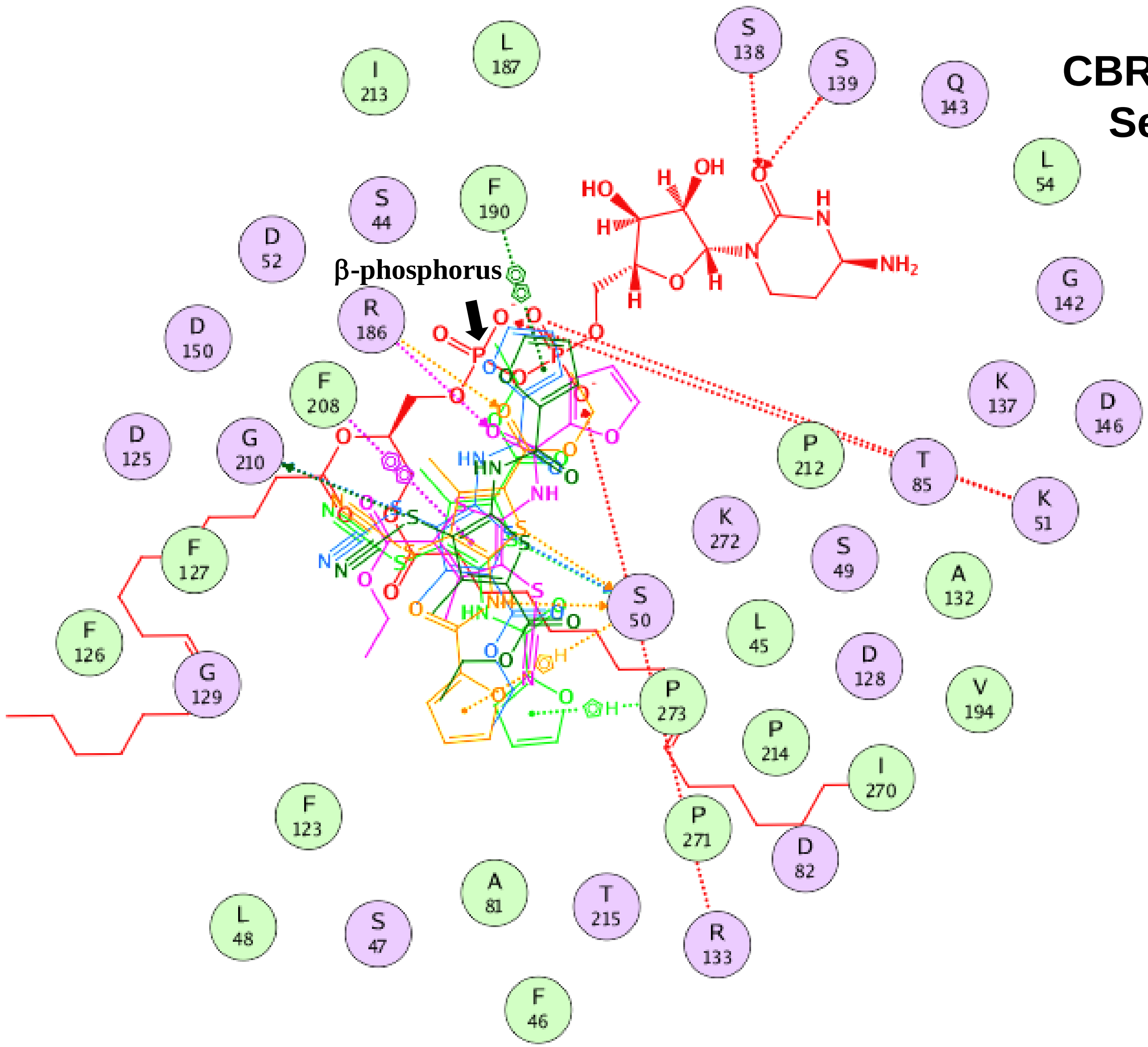

Figure S6. Overlay of ligand interactions between top five poses of CBR-5884 with *C. albicans* AlphaFold Cho1 model. The bound CDP-DAG is also shown and the β-phosphorus is indicated. Dotted lines indicate interactions. Docking scores MOE(S) of serine and CBR-5884 were shown.

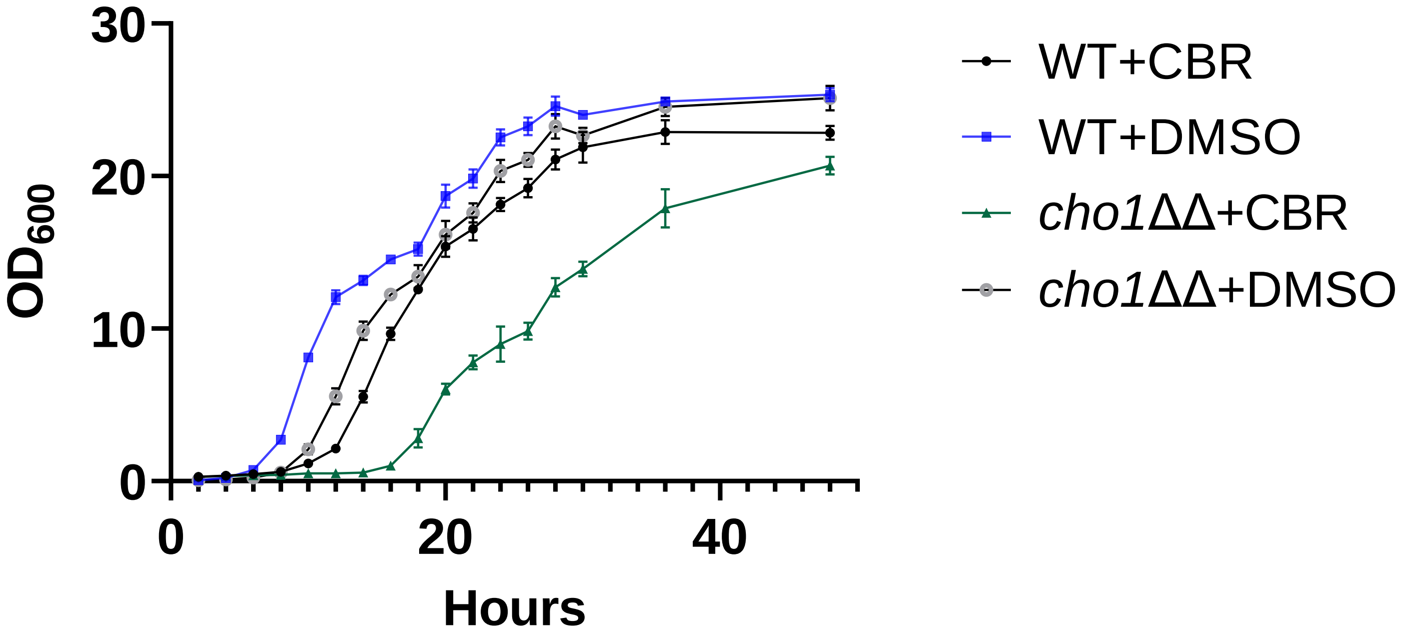

Figure S7. Growth curve of wildtype and *cho1*ΔΔ *C. albicans* strains in the presence of 170 μM CBR-5884 or equivalent DMSO in YPD from 0 to 48 hours. The dots represent the mean values of six biological replicates, and the error bars are ± standard deviation (S.D.) values
